# Spirit of the English Periodicals, and Notices of English Medical Literature

**Published:** 1835-07-01

**Authors:** 


### Periscope;

OR,

### CIRCUMSPECTIVE REVIEW.

" Ore trahit quodcunque potest, atque addit acervo."

### I.

### Spirit of the English Periodicals, and Notices of English Medical Literature.

### Catalepsy.

The following case has appeared in some of the Irish newspapers, and is copied into the Dublin Medical Journal, so that we take it for granted the editors have ascertained the authenticity of the statement.

" The subject of the disease is an extremely handsome female, about the age of nineteen, a resident of Rathdrum, county of Wicklow, and a married woman. About June last she came to Dublin for a severe hurt in the hip, and was at the time *enciente*. A variety of treatment was adopted during some time she remained in Dublin, and during which time she had very bad health, loss of appetite and rest, and great dejection of spirits ; but from her peculiar state, very strong or active remedies were considered inadmissible. She was unable to leave her bed ; but finding herself not getting better, her friends or husband not coming to see her, she suddenly got out of bed, dressed herself and walked about, though for some days before she had been unable to do so, or even at all leave her bed ; she also left the hospital, but returned to it in October last, with an increase of the disease for which she was first admitted, and during her sojourn in the country she had miscarried.

A few days after her return to the hospital she was greatly excited by the abuse of a drunken man, who spoke very harshly to her, and immediately after she got a very severe hysterical fit, which for some time returned daily, but about the ninth day she had a paroxysm of quite a different character, which was always very severe : all the muscles appeared to be firmly fixed—impossible to open either the jaws or hands—twists about her thumbs—and, should any person endeavour to hold her, she strikes at them with the greatest violence, catches at her hair, and, if not prevented, would tear it out in handfuls; she attempts to bite her own hands, and those of any person near her, and if prevented she bites the bed-clothes; her feet, if left loose, she kicks with dreadfully, in all directions. One of these paroxysms lasts but a few minutes at a time, but recur during the day very frequently, and are brought on by any slight disturbance. During the intervals she is apparently well, and in good spirits.

So far her malady may be considered of an hysteric nature, but when she was visited by the physicians on the morning of the 18th of October, it was found that a most singular and most inexplicable symptom had established itself during the night. It appears that she had experienced in the course of the night a paroxysm of unusual duration, which had commenced by an acute 'stitch' in her side. When the fit had passed away, the voice of the patient was utterly gone, and up to the time of committing this account to paper, she had not articulated a syllable. The sense of hearing, however, has remained unimpaired, and either by writing or by signs she makes it known that she understands any question which may be directed towards her. On the day after she underwent this loss of voice, the paroxysms commenced to assume a more violent aspect, and also to return with increased frequency. Soon after which change for the worse, her disease acquired for the first time all the features of a case of genuine *Catalepsy*. About a minute after the cessation of one of the fits, and when she appears to be perfectly recovered, she *suddenly* drops into what appears to be a sound sleep. The countenance is clothed with an expression of the purest placidity, the eyes are closed, and when, with a finger, the eyelids are raised, the eyeballs are seen to be rolled upwards, and the pupil dilated; she breathes quietly, and her arms, legs, and feet, though of themselves rigidly extended, are capable of being bent without any violence, and retain whatever position they may be placed in. At first the *cataleptic* seizure occurred only after the other paroxysms, but a few days only elapsed when it began also to precede them, and at the same time to extend itself to a greater length. On the 25th, the following was the order in which the series of very interesting symptoms exhibited themselves: the patient was in the act of making signs in reply to a question which had been put to her, when suddenly a smile overspread her face, and in an instant the cataleptic insensibility was completely established. She lay tranquilly for about fifteen minutes, when a violent paroxysm introducing itself as instantaneously as the first, interrupted her repose for about three minutes; then again the catalepsy returned, and lasting for another quarter of an hour, it left her in full possession of all her faculties except speech. She awoke, uttering a few piercing cries, which gradually subsided into a sighing moan, and then pressed her hands quickly to her left breast, as if she had pain in the direction of the heart. Another day that we saw the patient, the catalepsy lasted upwards of three hours, during all which time she lay extended and perfectly motionless; on this occasion, however, her countenance no longer presented its accustomed placidity; its veins, to their remote branches, swollen with blood, and her colour became of a dark purple; her limbs stiffened into unusual rigidity, and remained so until the dark hue deserted her face, when instantly they became relaxed and pliable. Whilst entranced in the manner described music was played near her, but she seemed not in any manner to notice it: her head was then placed over a tub, and cold water was discharged upon it from a height: she screamed violently, her face became suffused, and she had a fearful fit of sobbing, but did not remain conscious. On being questioned afterwards, at a moment that she was sensible, as to whether she had heard the music, or felt the shock of the fluid, she replied in the negative. It has been asserted in some medical publications, that patients in the condition of this girl were sensible of impressions made on the epigastrium, on the soles of the feet, and on the palms of the hands: it was accordingly attempted to excite her attention, or to arouse her by directing loud sounds to those parts, but the trial did not succeed. It is remarkable, that whilst all the more striking functions of life are suspended by these attacks, the circulation never betrays any other disturbance than an inconsiderable acceleration. The features of this case enable us to understand the so called 'Extacies,' in which cunning enthusiasts of several sects have represented their tools or their dupes to have been transported, 'as to their inward man, into the presence of the Deity, and there to have been favoured with special revelations.''*

---

GALVANIC EXPERIMENTS. By Dr. DUNBAR, of Winchester, Virginia.†

The following experiments are detailed in the first Number of our late contem-

* In a recent number of the Lancet, ERINENSIS has impugned the accuracy of the above report. But as this scepticism is mixed up with strong party-feeling, we do not deem it necessary to suppress the case, unless its authenticity be denied by more than anonymous testimony.—*Editors*.

† Baltimore Medical Journal.

porary, the Baltimore Medical Journal.

"Negro Ben, the subject of these experiments, aged 26, was a stout, well built subject, the muscular tissue of the body remarkably well developed, indicating great power. He was suspended from the gallows about thirty-five minutes, and in ten minutes after being cut down was delivered to a professional friend by the sheriff for me, and brought to a room (where every thing was arranged for the experiments) which had been liberally granted by the mayor, adjoining the town hall. The body was immediately placed on the operating table. The acid mixture (dilute muriatic acid) was thrown on the plates as soon as the conveyance bringing the body appeared in sight. It was discovered from the unusual freedom of motion in the neck, that it was dislocated —or, as it is called in common language, the *neck* was broken; and upon cutting down in a subsequent stage of the experiments, it was satisfactorily ascertained that the first and second vertebræ (atlas and dentata) were separated by a space sufficient to admit the end of the little finger. The appearance of the face was quite natural, and seemed as if the unhappy subject of the justly offended law had passed from life with none of those struggles and dreadful agonies which attend the throes of dissolution, and particularly in this most horrible mode of causing death.

The experiments were now commenced by a gentleman to whom was assigned the anatomical part, exposing, by a very handsome dissection, an important nerve in the neck, which supplies the nervous power to the lungs and stomach (par vagum, or pneumogastric.) A long silver needle, similar to those used in acupuncturation, was now introduced, through the space between the ribs, deep into the substance of the heart. The object of introducing a needle into the heart was to endeavour to ascertain whether any irritability remained, and to attempt to throw some light on a disputed question, whether the heart was susceptible of excitation by the galvanic fluid.

The positive pole of the battery was now applied to the nerve (par vagum), and the negative pole to the silver needle in the heart. There was no perceptible action of the heart, as it would in all probability have been evidenced by the quivering motion of the needle if it had taken place: and in no way that we could discover, was its irritability evidenced, either by the simple introduction of the silver probe, or the action of the galvanic fluid. But the effect on the other parts was very evident. The muscles of the neck and chest were affected by strong convulsive twitchings, most strikingly displayed in those muscles called by anatomists platysma-myoideus, sterno-thyroideus, and mastoideus, pectoral and intercostal muscles. There was also a convulsive motion of the muscles in the region of the stomach, and a contraction of the muscles of the throat, as in the act of swallowing.

A needle was now inserted into the tendinous head of the diaphragm, the positive wire of the battery applied to the par vagum nerve, and the negative to the needle. The result was a slight convulsive motion extending over the chest and abdomen. The contraction and relaxation of the diaphragm was very evident from the protruding and relaxation of the abdominal muscles; the effect seemed to increase as the acid had time to act on the battery. The positive wire was now applied to a needle inserted into the seat of the phrenic nerve (a nerve distributed to the diaphragm, and has an important influence in the function of breathing.) An incision was made down to the tendinous head of the diaphragm, and the other wire (negative) applied to the incision. The result was very similar to the preceding experiment, with the addition of an agitation of the chest, compared by an intelligent gentleman present, and a close observer, to that of a person affected by hiccough.

I think it proper to notice, at this stage, a peculiar action of the galvanic fluid on the nerve and muscular fibre, observed by myself, and confirmed by one of the gentlemen assisting me. The positive pole, whenever it touched the nerve or muscle, produced an action or whitening very similar to that which is produced by lunar caustic when applied to an exposed muscle.

A nerve above the middle arch of the eyebrow (supraorbital) was now exposed and the positive pole applied to it, and the other below the lower lid. The result was a contraction of the muscle, causing a natural wink, and an opening and closing of the eyelids, which was compared to that when the eye closes in a living person, from fear of being injured by some offending object being thrust at the eye. There was also a contraction of the muscles of the cheek, similar to what is seen in some persons who suffer from neuralgia of the face —or aptly likened by a friend to that motion of the cheek when an effort is made by the motions of the face alone to drive off an annoying fly which has settled upon it, without being at the pains to raise the hand for the purpose.

In the next experiment a silver needle was introduced into the facial nerve, one pole applied to it and the other to the cheek. The effect was slight motions of the face, and distention and contraction of the sides of the nostrils, resembling much the expression of disdain. The effect on this and the preceding nerve was very slight, compared with that produced by Dr. Ure, and so vividly painted by him. He says 'the expressions of rage, terror, anguish, and ghastly smiles were produced, and united their hideous expression in the murderer's face, surpassing the wildest representations of a Fuseli or a Kean.' In these experiments on the muscles of expression, with the exception of the expression of disdain, as before mentioned, none of those wonderful plays of the features were well marked which are so manifest when the countenance is animated by the mind, in its varying conditions of wild passion and pleasurable emotion.

The nerve going to the tongue (hypoglossal or 9th pair) was now touched by the positive pole. An interesting result ensued in the production of motion of the tongue alone. The positive wire applied to the silver needle inserted in the facial nerve, and the negative to the tip of the tongue, a very striking effect was produced, characterizing this as one of the most interesting experiments performed. The result was a rapid vibratory motion of the tongue, compared by several gentlemen to that of a serpent's tongue when alarmed or enraged. There was also a swelling or bulging out of the flesh or muscles under the lower jaw, which was agitated by a quick vibratory action, and one gentleman thought he heard the teeth striking together. The next experiment was the application of the wire to the muscles which assist in closing the lips and mouth. The result reminded me of the action of the lips when a person is muttering to himself, or about to utter words in a soft, low voice—or, in the words of a valued friend, extracted from the notes furnished me, the effect in his view in this experiment was as follows:—'The application of the galvanic influence in this experiment really did appear like animation; the lips acquired a motion precisely like those of a person reading to himself; and I do not know if this was not the most natural and pleasing experiment.' The expression of countenance of the criminal, in this stage of the experiment, caused a stare of amazement in many of the spectators.

A nerve (median) in the arm was now exposed, and an incision made into the middle of the little finger—positive pole applied to the nerve, and negative to the little finger. A most interesting and vivid display of the galvanic power was the consequence. The arm raised itself from a horizontal position with so much strength and violence, as to require the exertion of considerable power in the operator to restrain its freedom. It repeatedly made efforts resembling those of life to jerk its hand away from his grasp, and when permitted, struck against the chest with great violence, settling in the attitude of a pugilist when prepared to defend from the attack of an adversary. The motions of clenching and opening the hand, and the drawing up and extending the arm, during the progress of this experiment, was described by one gentleman something like the motion of one sowing grain in a field, if the body had been erect. The principal muscles of the arm were contracted so as to form a swelling, and their lines of demarcation were very conspicuous on the skin. During part of the time the forearm continued in its fixed position, and exhibited a tremulous motion, resembling that of the limbs of an animal immediately after receiving a blow on the head. The fingers were clenched on the hand in this experiment. These phenomena continued about half a minute, and then ceased. The poles of the battery were then removed, and soon after re-applied. The same phenomena ensued with equal violence upon the repetition of this experiment several times.

The ulnar nerve was then transfixed with an acupuncturing needle in the elbow, and positive pole applied to it, and negative to the little finger. The effect produced was a rapid motion of the fingers, but in a manner which was particularly striking to all who witnessed their action. Instead of being flexed at the same time, they moved sometimes rapidly, at others in a more gradual motion, but alternately, being greatest in the little finger, which was the most flexed, and diminishing towards the forefinger. The motion of the fingers, in this experiment, was compared by one to that when playing on the flute, and by others to the action of a violin performer when in playing he works upon the strings of that instrument.

These two experiments were decidedly the most remarkable, and exhibited the power of this most wonderful agent more completely than any other performed, and excited great and renewed surprise and astonishment at the effect of this magical influence, which could produce such wonderful phenomena, and so closely resembling those of a living person in the limbs of a body, whose spirit had deserted it for ever.

A needle was now introduced into the spinal marrow through the vacancy caused by the separation of the vertebræ, and another inserted into the heart and head of the diaphragm, but little effect was produced, other than the quivering motion of the muscles of the neck and chest. The spinal marrow being now completely exposed in the neck, positive wire applied to it and the negative to the foot, the effect was not remarkable; there was a slight convulsion of the muscles of the limb.

A needle being now inserted into the sciatic nerve, and another in the ham, there resulted a spasmodic action of the large muscles of the thigh; when the needle was changed to the inside of the foot, and the positive pole applied to the one in the ham, and the negative to that in the ankle, a much more marked and decided effect was perceived. The leg vibrated strongly (the subject lying on the abdomen); a swelling of the muscles of the calf of the leg; the toes were flexed, and extended with considerable freedom, at an angle quite as much as when extended in the act of stepping or walking.

With these the experiments ceased, the body having become externally cold, and the irritability sensibly exhausted; the power of the battery appeared also very much diminished, which will account for the weakened action produced in the last experiments.

I have already occupied so much space, that I am admonished to draw these remarks to a close, which I shall do in a few words. It may be proper to mention the power of the battery employed in the above experiments, and that of Dr. Ure, so that a comparison of results may be instituted, and it will then be left to scientific persons to judge of the success which has attended these experiments. The battery belonging to Mr. Edmondson, and used in this series of experiments, consisted of two hundred pairs of Wollaston's plates, each two inches square, arranged in four troughs, connected by tinfoil communications, but which, from a more perfect mode of insulation, was estimated in Dr. Cohen's letter to me to equal one of three hundred or three hundred and fifty plates, constructed in the usual manner. Dr. Ure's battery consisted of two hundred and seventy plates four inches square, with insulating handles. There was therefore a great difference in the surface exposed to the acid. In Ure's plates, a surface of sixteen inches was exposed to the combined action of two powerful acids, nitric and sulphuric; whilst in ours a surface of four inches only was exposed, acted upon principally by muriatic acid. During one stage of the experiments, when the action of the battery appeared decreasing, a small quantity of nitric acid was put in the troughs, which was just before those beautiful experiments on the arm.

In the performance of these experiments, the gentlemen associated with me were Drs. Conrad and Davidson, and my pupil Mr. Lees.

The medical gentlemen present at these experiments, were, of the town, Doctors Holliday, Baldwin, M'Guire, and Pennington—of the country, Doctors Lynn, Gray, and Orrick—and several students of medicine and scientific gentlemen."

---

### Spitting of Blood.

Much unnecessary alarm is sometimes occasioned by the issue of blood from the mouth, since the lungs or stomach are considered as the organs furnishing the vital fluid. A close examination occasionally reveals the source in the fauces, posterior nares, or the gums, when, of course, the alarm is greatly moderated, and the means of arresting the hæmorrhage rendered obvious.

Dr. Christian, of Dungarvan, has related an interesting case of this kind in the Dublin Journal for March last, of which we shall give a short account. A delicate lady, of middle age, awoke at five o'clock in the morning with what she called "spitting of blood." Some sulphuric acid was given without effect, and Dr. C. saw her at nine a.m. She was pale, but resigned—pulse frequent and firm—the quantity of blood lost was not great—but the decided opinion of all present was, that it came up by *vomiting*. The blood did not appear frothy and florid, as from the lungs, but was coagulated, and part of it dark. In neither of the basins was there any of the contents of the stomach. Superacetate of lead and opium were given, and no particular examination of the mouth or throat was made till the arrival of the family medical attendant. The mouth being then washed out, it was discovered that the hæmorrhage issued from the sockets of two of the lower incisor teeth, which were loose. The bleeding was easily arrested, after extracting the teeth. It is probable that part of the blood had been swallowed during sleep, and was actually vomited afterwards.

---

### Monomania.

An instructive case of this is narrated by Dr. Christian, in our Dublin contemporary for March of the present year.

A young unmarried female, aged 20 years, after experiencing some depressing circumstances, began gradually to entertain an unaccountable dread on the score of salvation. This increased to a miserable extent, and menstruation became suspended. A change in the hallucination now took place. She fancied that God had allowed her to become pregnant, as a punishment; and so strong was this idea, that no argument or reasoning to the contrary was of any avail. She ate well, slept well, and was able to attend to domestic affairs. The only symptom she laid any stress upon was the existence of a *sensation*, general in the system, but more especially in the abdomen. She could not describe it, or liken it to any thing. The mental distress was excessive. A draught of oil of turpentine, and ol. ricini was given, but without any beneficial result. As the period for menstruation was approaching, aloes and myrrh were given every night, and leeches were applied near the pudendum. Pediluvia, frictions, &c. were also applied. Under this treatment the menstruation returned; but there was no abatement of the hallucination. Camphor, valerian, assafœtida, and carbonate of steel were given, with frictions of antimony over the spine. In eleven days after menstruation, she had a release from the monomania. She again menstruated before the regular period—and became free from hallucination.

In the above case our author professes that he was guided by M. Andral in the indications, and by Dr. Graves in the pursuit of those indications. It is supposed by Andral that " the diseased perception points out the seat of the diseased action." But we apprehend that this rule will often fail to guide us. When the fear of damnation predominated in the foregoing case, where were we to look for the seat of the hallucination, except in the brain? When menstruation became suppressed, we had some tangible clue to go by. The restoration of the uterine function could do no harm, and might do good. After all, the *immediate seat* of mania and monomania must be in the organ of the mind, and the question next to be solved is this—does the brain suffer primarily or secondarily? Even in the foregoing instance we have no proof that the amennorrhœa was not consequent on the cerebral disturbance, and not the cause of it. The catamenial disorder succeeded the mental.

---

### Dr. Elliotson on Kreosote.

A short account of Dr. Elliotson's paper on Kreosote is given in a recent number of the Medical Gazette, from which we extract the following particulars.

" After some prefatory observations on the importance of attention to the medicinal properties of drugs and chemicals, as a source of light and power in the cure of diseases, the author stated, that having his attention called, early in 1834, to the therapeutic properties of kreosote by reports from foreign sources, he had begun his experiments by administering it to patients whose cases were ascertained to be unmanageable by other means. He first tried it in phthisis and epilepsy, and afterwards proceeded with the inquiry in cases of neuralgia, cholera, diabetes, &c. He usually began with two or three drops suspended in watery mucilage, and gradually raised the dose to ten, twenty, or more drops, until the stomach would no longer tolerate increase; over-dosing producing nausea, vomiting, headache, vertigo, heat of tongue, fauces, and œsophagus. He found the remedy quite powerless in phthisis, in whatever form, or dose, or mode exhibited. When respired, however, he occasionally observed temporarily increased facility of respiration and expectoration, as, indeed, might be expected; for it cannot be doubted that tar fumes have in some instances proved useful, as in the hands of Crichton and others. He mentioned that in some instances of bronchorrhœa, or chronic catarrh, he had obtained excellent effects from its inhalation from a solution of five to fifteen drops in about a pint of water, repeated several times daily.

In epilepsy he found little effect of any kind; but from an apparently tranquillizing power which he thought he observed in it in that disorder in some instances in irritable subjects, he extended the use of kreosote to hysteria, neuralgia, and other forms of morbid excitability.

Case I.—A young girl, labouring under acute pain in the hypogastrium and pelvis, with various other nervous symptoms, and habitual obstinate constipation. At first the pain, &c. recurred irregularly, but at length every morning between seven and eight, and lasted till night, after which she lay comatose until morning. Her face, gestures, &c. expressed extreme suffering. The evacuations healthy; bladder free of calculus. Every known remedy had been tried before her admission into St. Thomas's. Three grains of morphia relieved her a little every morning, but was soon discontinued as unavailing. July 22d, ordered a drop of kreosote thrice daily. The dose was gradually increased to seven drops. She improved rapidly, and left the house well in a month, having meanwhile regained her flesh and looks.

Case II.—A man, ill of neuralgia of the nares and palate three months, suffered dreadfully from pain, with sympathetic contortion of the features. He had been ill three years previously, and recovered without remedies. He began with three drops of kreosote three times a day, August 22d. On the 28th he was taking six minim doses, and was better, the pain recurring less frequently, and his sleep being much less disturbed by its paroxysms. The dose was raised at length to eighteen minims, and he soon got well enough to wish to leave the house.

The author noticed two other cases still under treatment, in which the disease seems nearly cured.

During these experiments the cholera broke out in the hospital, and it occurred to Dr. E. to try the tranquillizing power of kreosote in that *opprobrium medicorum*. In two cases it was exhibited with the effect of stilling the vomiting completely, but without other advantage; these cases likewise proved fatal. They suggested, however, further trial of its anti-emetic power; and the result of his experience was, that he knew of no medicine at all to be compared with kreosote in arresting vomiting, against which he had repeatedly known it succeed after prussic acid had failed. It has proved in his hands equally powerful to arrest vomiting when present, and to prevent it when threatening. In dyspepsia also, characterized by pain, acidity, nausea, &c. he has found it very useful; but he has observed flatulence aggravated by it.

He was led to try it in diabetes, from having accidentally read an account of a case in which it had been successfully administered.

CASE VII.—A country gentleman, of 60, ill four or five years with foul tongue, excessive thirst, and very saccharine urine, was ordered kreosote. On the 10th of September he was making water but seven times in twenty-four hours, instead of fifteen times as in August; his urine contained scarcely any sugar, and he felt himself quite well.

CASE VIII.—A medical man (Nov. 8), ill eight months, made twelve quarts of urine, of specific gravity 1038, per day. Stomach excessively acid; bowels costive. He took kreosote. November 25th, spirits, strength, and general health, greatly improved, and his amendment altogether surprising.

CASE IX.—A gentleman of 60, six months ill, making six quarts of water in twenty-four hours, of specific gravity 1037, took kreosote. Nine days after seen again; his health much improved. In December his thirst had disappeared, his urine come down to three pints, with proportional general amendment; but the urine still of specific gravity 1037. He has since been heard of, and continues to improve.

Such were the facts of the paper, the incompleteness of which, to a certain extent, the author pointed out, but accounted for satisfactorily from the rarity of some of the diseases, and the novelty of the whole inquiry."

---

### UNIQUE DISEASE OF THE HEART.

This case was observed by Dr. Hanna, and is related in the Dublin Journal for March last. The patient was 31 years of age, and of great bodily strength and activity. He had also lived freely. On the 25th August he received a severe fall on his back from a horse, and two days afterwards felt a beating at the heart, together with nausea. From this period onwards, he was subject to palpitations. He once heard something crack loudly in the middle of the sternum a little to the left side, succeeded immediately by a burning pain shooting occasionally under the scapula and down along the left arm. Afterwards he was seized with such severe palpitation, whilst hunting, that he fainted. He was relieved by blood-letting. When Dr. H. saw him, he was emaciated, and had dyspnœa, orthopnœa, palpitations, distressing dreams, cough, &c. pulse rather small, but regular. Between the second and third ribs, near the sternum " a loud whizzing rush of fluid was heard, with double beat." This was audible throughout the whole region of the heart. The impulsion was greater than natural. Percussion returned a clear sound. The disease made progress—the sense of suffocation was intolerable—and death took place. The pericardium contained a few ounces of clear serum, and that portion of it reflected over the heart was partially dotted with red spots, and presenting some sheets of lymph. The heart itself was twice or thrice its natural size. "On making an incision from the origin of the aorta along the ventricle to its apex, I opened into a cavity, which I at first conceived to be that of the ventricle, but soon finding my mistake, I removed the heart and took it home, where I proceeded to examine the cavity. It might contain a small orange, and was formed on the external parietes of the left ventricle. It was separated from the cavity of the ventricles by what seemed the inner coat transformed into a thick fibrous membrane, while on the outer wall, the muscular texture of the ventricle was quite effaced, as if by compression. It was lined with shreds of coagulable lymph which, easily peeled off. Just at the summit was seen a small, round, smooth opening, about two lines in diameter, leading at once into one of the sinuses of the aortic valve, and situated about four lines below the mouth of the coronary artery."

We agree with Dr. H. that the above was an aneurism of the aorta developed in a very unusual place—a lesion to which there is probably nothing precisely similar on record. The aneurism made its way into the paries of the ventricle, and there formed its nucleus or sac.

---

Oration delivered before the London Medical Society, March 9th 1835. By Walter Dendy.

We had not the pleasure of hearing this oration, when delivered *viva voce*, but we heard ample encomia passed on it, at the subsequent dinner of the Society. We now have it before us, and can safely say that it is a composition that does great credit to the talented and well-informed orator. Perhaps it may be remarked by some fastidious critics that the composition is too highly wrought—too full of tropes and figures—and abounding too much in erudite quotations. These, after all, are very venial faults—but in an oration, they are not only pardonable, but almost necessary. The matter, however, is that to which we look with most interest, and we think it exceedingly good. We shall make a few extracts to enable our readers to judge for themselves. After taking a rapid historical glance at the origin and progress of medicine, he thus alludes to "those points which tend to control, to foster, or to vitiate its influence."

"If we reason abstractedly, no one will be so bold or blind as to express his regret at the diffusion of knowledge; but it may be doubted if extreme facility of acquirement always administers to the benefit of society, if the profusion of lecturers whom the crowded ranks of the profession often drive to that source of emolument in default of practice, do not increase the evil they complain of, and commit a species of professional suicide.

I will not assert that in mechanics and in medicine there is an analogous law, that the gain in celerity or space is a loss of power, but at least there is in the latter this impediment—superfluity of numbers. Does the honey-bee seek its balmy treasure in those gardens where the swarm of locusts had alighted—the leaves and blossoms of which they had devoured? and will not this superfluity (although the schoolmaster, who is abroad, may brand my assertion as illiberal) blight the hope of remuneration for expenditure and laborious study?

Let me be understood. If the Profession were not glutted with embryo practitioners, if professional success bore an exact ratio to professional attainment, I would not venture to argue as I do. But is it not a fact, that the precincts of Bridge Street have been deluged by applicants, who jostle each other like lacqueys in a lobby, and rush with the ardour of an Olympic struggle, for the privilege of gaining—what? the post of professional drudgery, and that for a miserable pittance, compared to which the wages of the powdered coxcomb in the hall of affluence were a princely revenue. Can I heighten this picture? Yes, I will place before you the accomplished child of genius and of study, whom disappointment has rendered up a prey to melancholy and despair—his hope blighted—the mine of learning within him unexplored and useless—his life a blank. We may meet him, perchance with pale and haggard visage gliding slipshod through the streets of this metropolis, a beggar —or with a sensitiveness closely allied to genius, shrinking with instinctive dread from the disclosure of his penury, and at length, like Savage, and Otway, and Chatterton, (but more wretched still than these, for they had the golden rays of poesy to illumine *their* dying hour,) yielding his miserable life within the walls of a hovel or a prison. This is no exaggerated picture, it is sketched from the life; and yet, with these truths before us, there are some who would open still wider the door to these miseries, who even advocate unrestricted practice, opposing all attempts to raise higher the scale of professional education by which alone this mighty evil can be checked.

And is the proposition of the high curriculum a recent innovation? In so remote an æra as the eleventh century, the school of Salernum, founded by the Duke of Normandy, decreed a test of attainment as severe, according to the existing knowledge as now; and in the reigns of Maria Theresa and Joseph II., five years of study and severe examination were essential for the degrees of Doctor in Medicine or Surgery, the only legalized practitioners in Germany.

Then, the Pharmaceutical Association of Great Britain, enrolled in 1794, represented the necessity of a higher grade of education, but their bill was strangled in its birth. In adherence to the wish of this Association to appropriate to themselves all compound pharmacy, its principle was vitiated. Thus ran the preamble of their petition: "As the apothecary necessarily attends patients without any emolument but what arises from the profits of the medicines he may vend"—

By this preamble, which truckles to the system of remuneration for goods delivered, the vendors of physic excited the jealousy of opulent and successful rivals. This fallacy, this anomaly in principle, was the ruin of their petition; it emasculated the practical clauses of 1815, and I fear it will undermine the basis of any attempt at improvement, so long as an actual identity of the science and the trade is fostered by the profession, and winked at by the legislature."

The foregoing picture of the distress in which hundreds of medical practitioners are plunged, in consequence of the over-crowded state of the pofession, is scarcely exaggerated. We every day see such melancholy effects of a redundant medical population!

Mr. Dendy very properly denies that a high scale of education drives students to foreign schools. "A protecting duty against the importation of knowledge could only emanate from the barbarity of the dark ages, shrouding half our wisdom, and destroying those sacred links of fellowship, with which peace has united the scientific world."

We are unable to give more than one other short extract from Mr. Dendy's oration.

"From this point springs a subject which has been regarded with no slight suspicion, and which I, above all others, must approach with diffidence—the system of *levelling upwards*.

With sincere courtesy I ask this question—What is the present relative condition of the Profession? It is true that, in the nooks and obscure crannies of this metropolis, we here and there stumble on one of the ancient stanch apothecaries, whose shelves, like his of Mantua, are decorated with

> "Green earthen pots, bladders, and musty seeds,

> Remnants of packthread, and old cakes of roses."

But such an association is itself a libel on the present fame of the worshipful Company, which enrols among its members men, whose learning and whose wisdom would not quail in argument before a senior wrangler hot from Cambridge.

The surgeon, once the lowest grade of all, although no station has yet been granted him in the courtly order of precedence, has long since emerged from his chrysalis state.

The memory of Hunter—of Cline—of Home—of Abernethy, will live while science lives; for they have contributed to elevate the study of a mere manual art into a sublime and beautiful philosophy. And here I shall only echo the sentiment of all who hear me, if, for a moment, I lament with France the loss of him who was so lately one of her chief ornaments and blessings—Dupuytren. Whatever his eccentricities—his errors—his life was one unremitting course of scientific labour; and he has left a proof, more impressive than any the storied urn can record, of his ruling passion strong in death, in the splendid legacy of 500,000 francs, the endowment of a lectureship, and an asylum. Taught by similar bereavements of her own, Britain has learned to sympathize with all her sister nations; and with sincerity and regret she will bestow her eulogy and her tear over the grave of the illustrious Foreigner!

From the living lustres of surgery I will not presume to select; but for whom, I may ask, is the high scale of study more essential than for him whose practice is attended by extreme danger, and whose failures are so fearfully exposed to the ordeal of public scrutiny. The practice of physic is the sailing on a calm and summer sea—that of operative surgery on a dark and stormy water. The stigma and the penalty of *mala praxis* are fixed on the slightest errors of a surgeon, which his more learned brother escapes, for he is sworn on the Rubric to practise *tuto, cito et jucunde*, and happily the secrets of their prison-house are not often unfolded by the hepatic, the cardiac, or the pulmonary tissues.

Believe not that I speak thus in the spirit of invidious distinction, in prejudice of those Corinthian pillars of science which support and adorn the most learned college. This were to place one rank in array against the other, not in the pure and goodly fellowship of scientific ambition, but in the vain aspiration to mere *ceremonial* honour, the "whistling of a name." No: I will rather hope for that conciliation of the classes which shall destroy this many-headed Hydra of Distinction, which may constitute a fellowship not merely of this college or of that, but a fellowship of science—that the olive branch of peace may entwine round the staff of Æsculapius, and the broken shaft of Machaon.

Is this union of practice a speculative novelty?—No; I might revert for refutation to the reign of Henry VIII.—I might cite the instances of Winslow—of Harris—of Harvey. I may refer to the practice of more modern surgeons, and to the declaration of scientific physicians of the present day before the Parliamentary Committee; and, may I add, that such union would release us from the dilemma of the French monarch, who, being urged to place a barrier between the physician and the surgeon, shrewdly inquired on which side he should place the patient?

And is this equalization?—No. The tree of learning, like that of the forest, requires not a variety of strata in which to strike its roots, but its *branches* are not all of equal length—its flowers and its fruits not of equal beauty and flavour. Nor is equality of artificial growth; the manufacture of an edict or a law. These may confer title—they cannot ensure fame; for study and wisdom and age will still be honoured, still be sought for, though uncherished by courtly favour, though unadorned by the tassels of a college robe."

There is much truth in the foregoing observations, however they may be sneered at by the proud aristocratical members of the Profession. Those who scorn the idea of "levelling upwards," may live to see the day when they may acknowledge that it is a much better system than that of "levelling downwards."

---

OBSCURE PERICARDITIS—DILATATION OF THE HEART—ŒDEMA OF THE FAUCES, &c. By SAMUEL JACKSON, M.D.

"Mr. ———, aged fifty-four years, temperament sanguine nervous; previous health generally good; had suffered in former years attacks of acute inflammation of abdominal viscera; had been actively engaged in business for many years, and exposed to great mental anxiety; habituated to the moderate but daily use of spirituous drinks. In the commencement of January 1834, he returned from a fatiguing journey of twelve hundred miles, during which he was exposed to severe cold, especially in crossing the Alleghany ridge. From the state of the roads, the stages could not run, and anxious to reach home, he travelled across the mountains in an open mail cart. He suffered severely from the cold and violent jolting, the vehicle being without springs.

After reaching home, he complained of excessive fatigue, kept in bed for two days, took some medicine without advice, and resumed his accustomed pursuits. He kept about, complaining frequently of shortness of breath when ascending stairs, of being easily fatigued, and often sighed deeply. This last circumstance was attributed to mental causes, at the time, as his affairs were not in a very satisfactory state. On the 29th of January, I was requested to see him. The night previous he had been out on a visit, complained of being cold and fatigued when walking home, and in the morning felt too unwell to get up. I found him complaining of cough, with sense of oppression in the chest; he had no heat of skin; the face peculiarly pallid; pulse exceedingly feeble and frequent; the chest every where resonant on percussion; the respiratory murmur pure in every part of the chest, gave no indication of pulmonary affection; the action of the heart rapid, its sound feeble. A blister was directed to the chest, with a composing cough mixture.

The next day, 30th, he felt relieved; the oppression diminished.

*February 1st.*—Had passed a restless night; indescribable feelings in the chest; skin cool and shrivelled. Directed emulsion of assafœtida, with acetat. opii.

2*d*. Night again restless; skin cold, pallid, face waxy aspect; great anxiety; the chest again examined, furnished same pulmonary indications; the heart alone seemed affected; the pulse irregular, so rapid as scarcely to be counted; the sound of the heart feeble and indistinct; the organ appeared to labour; no morbid sounds; head remarkably clear; stomach in excellent state. In crossing the mountains he had used stimulants more freely than usual, and suspected his present exhaustion might proceed from their sudden withdrawal. The suggestion was adopted, and warm toddy, with carb. ammoniæ prescribed. 12 M. Same state. 3 P. M. Has rallied in some measure; skin warmer, and feels more comfortable. 8 P. M. Skin still warmer, dry; pulse has more force. Continue stimulants.

3*d*. Night more tranquil than preceding; had one sinking spell, almost approaching to fainting; examination of the chest presented some indications as before, both as to the lungs and the heart; has a sense of extreme prostration and debility. When lying with his head low, the face is purple, from the stagnation of the venous circulation; when it is elevated, it immediately becomes pallid. The veins of the extremities very much distended, and cannot be emptied by pressing upwards along their course. Senses and intelligence are perfect; stomach in excellent condition; bears the stimulants administered every half hour perfectly well; sinapism applied along the spine; blister over the region of the heart, and on the lower extremities; calomel gr. i. every hour. No change occurred during the day. When the stimulants are withheld, the sense of sinking and disposition to fainting comes on. The stimulants were administered every half hour. No other effect apparent than to obviate the tendency to fainting and sinking. At 10 P. M. Tinct. opii, gtt. xxv.

4*th*. In the night had one sinking spell: this morning pulse rapid, irregular, and without force; hands purple; respiration made with considerable muscular effort; had a natural evacuation in the night; tongue moist and clean; sense of extreme exhaustion; mind clear; had the morning papers read to him; stimulants continued, with nourishing soups. 5 P. M. Had during the day several fainting and sinking spells; is then covered with cold sweats; pulse scarcely perceptible. The following mixture prescribed:—Sulph. quiniæ, gr. xij.; Elix. vit. ʒss.; Syrup zingiberis, ℥ss.; Aq. fluvial, ℥iss.; one drachm every hour. Stimulants continued. 10 P.M. Appears to have rallied in some measure; fingers less purple; pulse more distinct.

5*th.* A more tranquil night; has a feeling of more force; circulation improved; had the newspapers read to him this morning; is constantly in semi-erect position supported by pillows; stimulants diminished one half; nourishing soups. 10 P.M. Has continued in better state during the day.

6*th.* Disturbed in the night by a fire that occurred in the vicinity; was much agitated, and lost his sleep; the disposition to sinking and fainting was renewed and increased; stimulants again resorted to and increased; had three passages during the day, each perfectly natural; rallied in the day, and had augmented thirst; stimulants withdrawn entirely, and small pieces of ice held in the mouth to allay thirst.

7*th.* Rested well; has more force; has used no stimulants during the night; tongue has become red, with white fur; thirst increased. In evening prostration; respiration laboured; pulse fluttering; solution of sulph. quinæ and toddy renewed. 10 P.M. No improvement; carb. ammoniæ and wine-whey.

8*th.* Restless night; but little change; tongue moister; had three stools in the night, all natural; pulse small, feeble, irregular; veins turgid with blood that cannot be forced along them; respiration hurried, and irregular; stimulants continued; frictions on the spine, with ol. succin. ℞. Spts. camph.; ol. terebinth. and ol. succin. m. j. every two hours.

9*th.* Tolerable night; symptoms nearly the same; has appetite; stimulants withdrawn, except spts. Camphor, m. ij. every two hours. Evening. In same state.

10*th.* In the night became hoarse, with feeling of soreness in the throat; an obstruction there causes difficult respiration, and nearly prevents swallowing. On examining the throat, the whole fauces were tumid; the uvula swelled and thickened; the velum and soft palate pressed down like an inflated bladder; very slight redness; skin warmer than it has been, and pulse possess more force. Sinapised poultice applied to the external surface of the throat; gargle of infusion of Cayenne, and of a solution of iodine alternately used every fifteen minutes; punctured the swelled and tumid velum; a thin fluid oozed from opening. 1 P.M. Difficulty of respiration and suffocated feeling increased; applied saturated solution of nitrate of silver to the fauces, and again punctured the swelling; swallowed some wine gruel with tolerable ease, and had appetite. 4 P. M. Swelling of fauces greatly augmented; respiration suffocating. The danger of suffocation had become so imminent I sent for a surgical friend to perform tracheotomy as the only resource, and in the mean time punctured freely the tumour, filling up the fauces. A thin bloody fluid issued in large quantities, but without relief. Suffocation progressed every instant; longer delay was inadmissible, and with no other instrument than a pocket scalpel I attempted the operation. On making the incision through the skin, a thin bloody serous fluid was found existing in the cellular tissue, from which it discharged in a copious stream. A small opening only was accomplished in the trachea, into which the infiltrated cellular fluid was sucked in inspiration. Suffocation was completed, the patient's head fell over, the face bloated and blackened, and in a moment he expired. Half an hour only had elapsed since I had entered the room, so rapid had been the progress of the œdematous effusion."

The body was examined twenty-four hours after death, in the presence of

Drs. Jackson and Godhard. The anterior portion of throat presented a mulberry red colour, and, on making an incision along the front of the larynx, a copious flow of dark bloody serum took place. This effusion extended deep about the parts—viz. the larynx and neighbourhood. On examining the thorax, the pericardium was found glued to the diaphragm, and contained about two ounces of straw-coloured fluid. It was lined by a false membrane, one or two lines in thickness, in some places. The surface of the heart was rough and of a yellow colour, covered also by a false membrane. The right auricle was considerably dilated, and its parietes thickened. Several small tumours were found between the musculi pectonati, varying from the size of a pin's head to that of a large bean. They were attached either by a narrow pedicle, or by small cords, and, when cut open, were found to consist of a reddish grey semi-fluid mass, resembling disorganized blood. The capsules seemed organized. The right ventricle was filled with a black coagulum extending into the pulmonary artery. In this cavity also was found a group of the same kind of tumours. The left auricle was dilated and its parietes thickened. No tumours in this cavity. The left ventricle was dilated without hypertrophy, and in its cavity some of the tumours were also found, one of which was as large as a walnut. The lining membrane of the aorta was covered, to some distance, with a layer of coagulable lymph, half a line in thickness, and of recent formation. The subjacent tissue was highly inflamed. The pulmonary artery presented the same appearance. The abdominal viscera were sound. The brain was not examined. We give the following observations and reflections of the narrator.

"In the foregoing case a circumstance of interest also was present. Acute pericarditis existed, as was ascertained by the autopsy. But during life it was manifested by no positive diagnostic signs. The irregularity of the contractions of the heart existed wholly independent of pericarditis. I saw a gentleman in a consultation visit this spring, whose heart acted in the most irregular manner, with very feeble contractions. He died soon after quite suddenly from apoplexy. The pericardium, whose inflammation I had suspected to be the cause, was reported to me to be healthy, but the substance of the heart was softened, and an ulcer existed in its parietes.

In this case no fever, or acute pain, the common attendants on acute pericarditis, were present. The mind too so often disturbed with agitating fears in that disease, was perfectly calm and tranquil. From the obscurity of the symptoms I felt entirely at a loss to determine the true diagnosis of the affection. A cardiac lesion was evident, and a difficulty in the course of the circulation was apparent, but the precise nature of either could not be determined.

On a review of this case, I feel at a loss in deciding whether it would have been a preferable course to have attempted blood-letting for the relief of the circulation, notwithstanding the strong evidences of debility, instead of stimulating. From the apparent effects of the stimulants, they were indicated. The symptoms were lightened, and before the effusion occurred, a very positive amendment had taken place. But was not the effusion one of the effects of the stimulants? Yet why should their action be so local as to affect exclusively the throat? These questions it is difficult to solve.

Aortitis also existed in this case, yet no signs were present to indicate its existence. The pulse in this form of disease is usually tense and hard. The irregularity and feebleness of the heart's action may have controled this symptom, usually produced by arteritis.

The immediate cause of death appears entirely unconnected with the cardiac lesions. The symptoms originally present, and indicative of disease in the central organ of the circulation had been yielding; a decided amelioration had taken place, when without any assignable cause, the œdematous condition of the fauces and neck, ensued, extending to the larynx.

That the cause was local is evident from the œdema having been limited. In the external cellular tissue the effused fluid was deeply coloured with blood.

I have met with several cases previous to this, of œdema of the fauces of a lighter degree, and producing very suffocative respiration, showing its extension to the glottis. They all recovered. This is the first instance I have seen of the disease in a fatal form."*

---

The Sphygmometer; an Instrument which renders the Action of the Arteries apparent to the Eye, &c. By Dr. Julius Herisson. Translated from the French, by Dr. E. S. Blundell. Octavo, pp. 46, 1835.

The profession is well aware that we were the first in this country to hail the stethoscope; and the event has not done discredit to our penetration on this point—however dull we may be, in general, as to our perception of the useful or ingenious. We pledge our reputation for sagacity, that the sphygmometer is one of the most silly and ridiculous baubles that was ever attempted to be foisted on the attention of the profession. A complicated apparatus to be fixed on the arm, or on the chest, to indicate the action of the heart and arteries—an action that will vary from Alpha to Omega while the apparatus is being applied, and which, after all, will not convey one-hundredth part the information to the *experienced* practitioner, which the finger will indicate! To the *inexperienced* it will only prove an ignis fatuus, and lead him into "sloughs and ditches." We will not insult the good sense of our readers, or waste our own time, in any attempt to break this butterfly on the wheel of criticism. The following description of the *apparatus*, which the English translator has added to the original Gallic bauble, will be quite sufficient for its exposure.

"The Apparatus must be made as light as possible, either of silver, ivory, or steel; it acts somewhat on the principle of a screw-pincushion, which it slightly resembles in shape; it is fixed upon the arm, in the same manner as the pincushion is made fast to the table, viz. by a spiral screw; this screw has upon its point a tabular circle, which being free, moves independent of the screw, and allows the latter to be regulated without disturbing the former, when in contact with the wrist, whilst the instrument is being adjusted over the artery.

The Sphygmometer is passed through an orifice or slit, prepared for its reception at the end of an arm, of an elliptical form (being round at the upper, and quite flat at the under surface), to prevent it from turning round;* this arm slides horizontally into an aperture, which is adapted with the utmost nicety to receive it, and possesses the advantage of being adjusted at any point to which it may be required, by means of a small screw placed vertically over it, which, when turned, firmly fixes the arm of the Apparatus into the aperture, and prevents it from advancing or receding, except at the will of the operator.

That part of the Apparatus containing the aperture, into which the horizontal arm slides, is placed perpendicularly, and is made to connect the lower part of the Apparatus with the upper by a spiral screw, which, when turned, either increases or diminishes the distance between the base of the Sphygmometer and the tabular circle."

Whoever has visited the Falls of the Rhine must have been very much amused by an exhibition on the banks of that noble river, which shews him,

---

* American Journal of the Medical Sciences, No. 30, Feb. 1835.

* "The first idea which suggested itself to me was, to have the Apparatus made on the principle of a Bracelet, but the great difficulty of keeping the Sphygmometer in a vertical position induced me to abandon it."

on a diminutive scale, and by means of a complicated apparatus, the majestic fall of water in a chrystal mirror:—This, too, when he has only to throw up the sash of the window, and see the original, in all its glory, within five hundred yards of him!! Such a Liliputian display, however, is philosophy itself, compared with the sphygmometer—a complicated piece of machinery for *seeing* the pulse, when it can be *felt* by the finger—the touch in this case, as in many others, being a thousand times less deceptive than the sight. But enough of the bauble.

---

CORONER'S INQUEST ON AN INFANT WHOSE HEAD WAS OPENED TO SAVE THE LIFE OF THE MOTHER.

A very disgraceful scene of this kind recently occurred in the Sister Isle. A female, Mrs. Fin, was in labour two or three days, under the care of a midwife—Mrs. Goolding. At length Surgeon Hayden, of Dublin, was called in, and finding the woman sinking, without prospect of delivery either by Nature or the forceps, proposed embryotomy. The husband would not consent for a long time, but at last did consent, and the mother's life was saved. It appears very clearly, from the minutes of a coroner's inquest, that some jealous professional neighbour, whose name we shall pass over, instigated the said inquest, by circulating unfounded and malicious reports, injurious to the character of Mr. Hayden. The unworthy object was not attained, and the disreputable motives were signally disappointed. The jury brought in a verdict highly gratifying and honorable to Mr. Hayden—first, that the child was, most probably, dead before the operation was performed—and secondly, that, had it been otherwise, the operation was absolutely necessary to save the life of the mother. They also attested to the skill, humanity, and judgment of the traduced member of our profession—Mr. Hayden. We hope this will be a lesson to those narrow-minded and invidious individuals, too numerous in the ranks of our profession, who would not hesitate to ruin the reputation of a contemporary to gratify their own spleen and malice.

---

CURIOUS CASE OF PARAPLEGIA. By Dr. WADDELL, of the United States.

Mr. H——, a distinguished lawyer of Athens, Georgia, died in the month of October, 1830, affected with paraplegia. He was rather tall and spare in person, pale in complexion, phlegmatic in temperament, and at the time of his death about thirty years of age. None of his family, ascending, descending, or collateral, have ever been peculiarly disposed to hereditary disease; indeed they are said to have enjoyed remarkable immunity from all kinds of chronic disorder. Before his marriage, about the year 1823, he was studious and sedentary in his habits; after that event he became more active.

It was about this time that he began to complain of cardialgia, nervous headache, pain at the point of scapula, and possibly some other symptoms of derangement in digestion. Two or three years afterwards, whilst on the circuit, attending one of the upper county courts, he slept in very damp sheets, and had all these symptoms highly aggravated. From this time onward, dyspepsia and hypochondriasis, with their ordinary accompaniments, made silent but constant progress. In April, 1829, he found himself somewhat wanting in ability to void the contents of his bladder; and this was the first symptom of paralysis. It may be proper to remark, that hitherto his bowels had acted well, and that he was not at all troubled with pain, either in the epigastric or hypochondriac regions. In August he first remarked that he was losing sensation in the soles of his feet, and this was followed by a similar loss in the calves of his legs, numbness of the thigh, and weakness of the lower limbs generally. Being a candidate for the state legislature, he attended the election at the County Court House, seven miles from home, about the first week of October. Here his strength was much exhausted by the exercise, and the excitement which probably attended on a successful canvass. Riding home on horseback the next day, he was seized with pains in the lower part of his spine, and spasms in the neighbouring muscles. He now soon lost sensation and motorial power in both of his lower extremities to a great degree. The temperature of his diseased limbs was variable.

Just at this period, the author of this essay was requested by his family physician to see him. He had been taking some aperient and alterant medicine, and no particular change being instituted in the treatment, he shortly determined on being carried to Milledgeville, a distance of seventy miles, to meet the State Medical Board as a council on his case. On his way, an application of tartar-emetic ointment was made to his legs, which produced very angry effects, and threatened something like gangrene.

Being arrived in Milledgeville, he underwent a course of treatment for fever, with which he was attacked, and had two issues established in his back by the council called to him. This was followed by immediate melioration of his symptoms, and onward from that time, an indefinite number of issues were kept discharging until within two months of his death.

His absence from home amounted to five weeks, at the expiration of which time he was brought back, apparently much improved in general health. His appetite in the course of a week became very vigorous, soliciting food of the strongest kind. Some indulgence of this propensity invited a return of his fever, but no symptom of moment appeared until February of 1830, when he was seized with universal muscular spasms of a very severe character. Colic, throughout the whole of his illness, was one of his most distressing symptoms, and this was accompanied by a great evolution of gas, and so much spasmodic uneasiness, as to threaten the extinction of life. For this he commenced the use of laudanum as the Spring advanced, and gradually increased its dose until within a few weeks of his death in October, at which time he would take as much as two ounces and a half in 24 hours.

A visit to a chalybeate spring in May, did not materially benefit him. Great emaciation of the lower extremities, together with permanent tonic contraction of the flexor tendons of the toes came on, and paralysis gradually extended upwards until July, when sensation became dull as high as the xiphoid cartilage in front, but not so high on the posterior part of the trunk. During the Summer his bowels were irregular; the power of the sphincter ani was lost, and also the sensibility of the rectum, as high as the sigmoid flexure of the colon. He received intimation of an approaching alvine discharge, by a sensation in his bowels similar to what might be expected from warm water passing through them. He sometimes had power to retain and void the contents of his bladder ad libitum; at other times all control of the vesical sphincter was lost, and the urine was discharged involuntarily. It was sometimes bloody, at other times it came away mingled with long shreds of mucus; during the latter part of his life, it consisted in a great measure of pure blood.

His treatment under the care of Dr. Linton, for the last two months of his life, consisted in drying up his issues, regulating his bowels with sulphate of alumine, calcined magnesia, &c. &c. Infusion of hops was substituted for tincture of opium, and the latter article was withdrawn entirely, so far as practicable. Moxibustion was instituted on his spine, feet, and knees. To the use of the latter remedy, there succeeded a development of very obscure sensation in the sole of one foot, and in the ankle of the opposite limb, together with a perceptible relaxation of the flexor tendons of the toes.

Three weeks previous to death, a pain commenced in the right hypochondrium, attended by some spasmodic movement of the corresponding external part, which was intermittent in character. Seventy-two hours before death, there issued from his bowels a profuse sanguineo-purulent discharge, followed by great prostration of vital power, inability to swallow, dimness of vision, and death, in the entire possession of every intellectual faculty.

The body was examined in the presence of Drs. Linton, Jones, Tinsley, and Waddel. The body was exceedingly emaciated. The bones of the spine were sound—as were the spinal marrow (lower part) and its investing membranes. The stomach was partly filled with a fluid resembling dark blood, but tinged with cystic bile. The mucous membrane was inflamed, as was that of the intestines, over which a quantity of loose purulent matter, of a yellow hue, was found floating. This proceeded from a perforation of the duodenum, about half an inch in diameter. The heart was remarkably small in size; but there was nothing else remarkable in the thorax.

The narrator apologizes for the imperfection of the foregoing dissection. They had not time or permission to examine the head. The author goes on to raise a theoretical edifice on this case, which never can stand against the slightest breeze of correct induction. He *imagines* the head was not affected, because there was no disturbance of the mental functions—and he *imagines* that the sympathetic nerve was the seat of the paraplegia, or at least of its cause, though said nerve was never examined. If we must rest on conjecture, where actual examination ought to have obtained, the great probability is, that the seat of the disease was in the head, because it has been found there often in cases of paraplegia, according to the testimony of Baillie, Abercrombie, and others.

---

### MEDICAL TUITION.

The following circular has been issued by the principal teachers of Bartholomew's Hospital, addressed to their colleagues in the British metropolis.

"The undersigned medical officers and teachers of St. Bartholomew's Hospital beg to address their colleagues in other hospitals and schools, on a subject of paramount importance to their common profession.

The immense advantages which London possesses, for scientific and practical instruction in medicine and surgery, have rendered it the great resort of British medical students; hundreds of whom repair every year to the hospitals and lecture-rooms of the metropolis, in search of that information which is to qualify them for the exercise of an arduous profession.

It appears to the undersigned, that neither the profession nor the public derive the full benefit of these advantages, under the present plans; and hence that it is expedient to revise the method of proceeding, in order to the adoption of such changes and regulations as may render the course of medical study more efficient.

The whole business of medical education, so far as lectures are concerned, is transacted within seven months—from October to April inclusive. During the same period, the majority of students attempt to accomplish also the important object of practical study at the bed-side of the sick. Two sessions are usually devoted to medical studies in London.

The consequence of these arrangements is, that so many courses of lectures on the various branches of professional knowledge are going on together, as to fatigue the attention and confuse the mind of the student, leaving him no opportunity for more than a casual and interrupted attendance in the wards of the hospital.

This evil would be considerably lessened if the five months that remain after the close of the medical session were devoted to practical study; which might then be pursued without interruption. But it is the custom for students to leave London when the lectures are ended, and not to revisit it till the beginning of the following session.

It must be obvious, that under such a system, in which the whole course of efficient instruction, both oral and practical, is crowded into the inadequate space of fourteen months, the best objects of medical education never can be attained.

The undersigned beg to propose for consideration the following plan :—

1. That the medical session should consist of ten months—from October to July, inclusive; leaving August and September for the vacation.

2. That the medical session should be divided into two equal periods, and that an equal distribution of the lectures should be made between these.

The hospitals would be open to students throughout the year, and clinical instruction would proceed constantly. The spring and summer being the most favourable time for attention to hospital practice, this portion of the year would probably be thought most appropriate for lectures on practical subjects.

The undersigned are aware that a change so extensive as that now proposed, could not be adopted by any single school without great risk of failure. They therefore recommend the subject to the consideration of the physicians, surgeons, and teachers of other hospitals and schools, with the hope that some common plan may be devised for rendering the system of medical education in London more suitable to its great objects.

(Signed) P. M. Latham, M.D.
George L. Roupell, M,D.
George Burrows, M.D.
Hugh Ley, M.D.
Frederick Farre. L.M.
Wm. Lawrence, F.R.S.
Henry Earle, F.R.S.
Edw. Stanley, F.R.S.
Thomas Wormald.

St. Bartholomew's Hospital,
April 13, 1835."

Advocating, as we have always done, an extension of the curricula of medical education, as the surest means of enhancing the character of the profession, and promoting the weal of the public, we need hardly say that we approve of every measure that tends to this object. But we do not think that the foregoing extension of the medical session is sufficient—though certainly an improvement on the present plan. Three years at least should be dedicated to the elementary study of so difficult a profession as ours, and we hope soon to see a regulation that will enforce even a longer range of study than that. We understand that the Apothecaries' Company have actually agreed on a regulation of this kind, with a similar, or nearly similar extension of the academic session. We do not participate in the apprehensions entertained by our contemporary, the Medical Gazette, that this new edict from Blackfriars may, "by raising the standard so high, incur the risk of placing the commodity beyond the reach of the less opulent." The poor will always have plenty of medical practitioners to attend them gratuitously—were it only on account of acquiring practical information. But even if we granted that a few should suffer from want of medical relief, (which, however, is a mere gratuitous supposition,) is that any reason why the standard of medical education should be below the proper level? How many thousands daily suffer from this cause at the present moment! Make the article *good*, say we, even at the expense of its being somewhat dear. Better that a few should have no physic at all, (of which there is little to fear,) than that all should swallow bad physic.

---

### Mirage.

" Nowhere (says Lieut. Burnes) is that singular phenomenon, the *Mirage*, or *Surab* of the desert, seen with greater advantage than in the Run, (near the Indus.) The natives aptly term it smoke; the smallest shrubs at a distance assume the appearance of forests; and on a nearer approach, sometimes that of ships in full sail, at others that of breakers on a rock. In one instance I observed a cluster of bushes, which looked like a pier, with tall-masted vessels lying close to it; and on approaching, not a bank was near the shrubs to account for the deception. From the Run, the hills of Cutch appear more lofty, and to have merged into the clouds, their bases being obscured by vapour.

The wild ass is the only inhabitant of this desolate region; they roam about in flocks, 'scorning the multitude of the city, and make the wilderness and barren lands their dwelling.' Their size does not much exceed that of the common ass, but, at a short distance, they sometimes appear as large as elephants. While the sun shines, the whole surrounding space of Run resembles a vast expanse of water—the appearance it commonly assumes—and which is only to be distinguished from real water by those who are long habituated to such visionary illusions. When the sun is not shining, the Run appears higher at a distance; but this has been remarked of the sea, and other extensive sheets of water, and is also to be accounted for in the deception of vision."*

---

### Intussusception with Stercoraceous Vomiting, successfully treated by Inflation. By J. Wood, M.D.

Mr. G., aged thirty-five, a type-founder, of nervous temperament, was suddenly seized, on the night of September 17th, with violent pains in the umbilical region: has before suffered two slight attacks of rachialgia. A medical gentleman in the neighbourhood saw him, and prescribed purgatives and hot fomentations. My attendance was requested the next night. He had obtained no relief from the remedies administered during the day; stomach had rejected them. No dejection for three days; frequent retching; little intermission from pain; pulse small and regular. Superficial examination of the abdomen detected no peculiarity. An acquaintance with his previous attacks led to the presumption that the present was painter's colic: he was, therefore, directed to take a pill containing two grains of opium, and one drop of croton oil every two hours till relieved; and to have an enema every four hours, and bags of hot sand to the abdomen.

*Sept.* 19*th.* Has taken all the pills: less pain through night, but since daybreak paroxysms have returned with greater force and frequency, accompanied by violent tormina and tenesmus; dry retching, especially after taking drink; hiccup; slight but general tenderness of abdomen; pulse irregular, irritated; no fæcal evacuation, but discharged in the course of the day a little bloody mucus, and vomited, towards night, a large quantity of green bile mixed with feculent matter. After a careful examination, the precise point of obstruction was ascertained. At first, only an unusual fulness and firmness in right iliac region could be discovered: but the hand lying upon the spot, a paroxysm of pain occurred, and an elongated tumour was felt to rise with an erectile motion: immediately there followed a gurgling rumbling noise, and a rush of fluid downwards against the point of obstruction. After two or three surges against the tumour, the fluid receded towards the stomach; but again returned with renewed violence, until the spasm subsiding, it passed upwards, the bowels taking on an inverted action. The treatment adopted was copious bleeding, the administration of purgatives, as calomel, infusion of senna, castor oil, enemata of tobacco infusion, hot anodyne fomentations, and at night large doses of opium.

20*th.* Aggravation of all the symptoms. In the afternoon Dr. Morril saw him, and after a careful examination of the case, and an acquaintance with the treatment pursued, he agreed in opinion that there were no grounds for hope. As a last resource, however, he proposed inflation of the bowels with atmospheric air. Placing the patient on his right side, the pipe of a pair of bellows was introduced into the rectum, and inflation cautiously began. This succeeded but partially, owing to the imperfection of the instrument; yet to our surprise, he proclaimed himself much easier, and was irresistibly driven to the commode, where he passed a large quantity of air and a gill of

---

* Vol. I. p. 318.

very fœtid, bloody water. A more perfect pair of bellows having been obtained, the tube was again inserted, and inflation employed till the abdomen became tense. He had no recurrence of violent pain after first inflation. Directions were given him to remain quiet in bed, and resist the disposition to evacuate the bowels, to take one drop of croton oil every two hours, and at the end of four hours an enema of mucilaginous fluid. He was visited six hours afterwards; had had no return of pain since inflation: within last hour two copious dejections.

21*st*. Had seven dejections since last visit; quiet sleep for three hours; somewhat feverish, thirsty; countenance anxious; pulse 125, small; tongue red, dry in centre. Full vesication from blister applied yesterday. Pretty severe enteritis followed, which yielded to the usual remedies in the course of fourteen days.

The result of this case was extremely gratifying. With regard to the treatment finally employed, we had no very sanguine anticipations of success; yet it must be admitted, that there is more philosophy in it than in many other plans adopted to lessen the ills of life. Of the numerous enemata administered in the forty-eight hours preceding the inflation, but a small quantity was retained, and that but momentarily. The use of them was persisted in with the hope of effecting mechanically the dilatation of the gut. In similar cases, the enemata cannot be thrown up to any amount: what is injected immediately returns. The effect is totally different when air is used. Its levity, its freedom from all irritating qualities, its elasticity and expansibility, give it a decided preference over the enemata. The nature of the difficulty, also warrants the view of its utility. The tendency of the peristaltic motion and of the ingesta, is from above, downwards; most cases of intussusception, therefore, are *progressive*, few *retrograde*. If then, we can dilate the stricture, the invaginated portion will escape from below upwards, and thus will be restored to its original situation. If the above explanation be correct, what remedy can compete with inflation? It certainly merits a trial, and is earnestly recommended to the consideration of the profession.—*Boston Medical and Surgical Journal and Med. Mag. Dec.* 15, 1834.

---

### Prejudices against Fish.

It appears from Lieut. Burnes, the author of very interesting travels in the East, that some of the oriental nations, and especially the Sindians, entertain a very unfavourable opinion of fish, as an article of diet.

"The natives of the neighbouring countries, and the higher class of people in Sinde, have a singular notion regarding the fish diet of the inhabitants. They believe it prostrates the understanding; and, in palliation of ignorance in any one, often plead that 'he is but a fish-eater.' The lower order of the Sindians live entirely on fish and rice; and the prevailing belief must be of an old date, as they tell an anecdote of one of the Emperors of Delhi who addressed a stranger in his court with the question from whence he came: he replied, from Tatta; and the king turned away his head. The stranger, recollecting the prejudice against his country, immediately rejoined that he was not a 'fish-eater.' I am not prepared to state how far a fish diet may affect the intellect of the Sindian, but I certainly remarked the prolific nature of the food in the number of children on the banks of the Indus."

We are inclined to think that freshwater fish, upon the whole, is not so salutary as fish of the ocean; and therefore there may be some foundation for the opinion, if not prejudice, of the natives of Sinde. Lieut. Burnes, however, has fallen into a very common error respecting the prolific nature of fish as an article of diet. It is true that there are more children to be seen in fishing than in inland towns. But this is owing to more early marriages in the former than in the latter. The causes, again, of the early marriages in the fishing-towns, are the greater facility of providing for families. The children can there be turned to some account, almost from infancy, in the various avocations connected with the making of nets, drying of fish, &c. &c. &c—circumstances not attendant on agricultural or even manufacturing localities.

---

### Œsophagitis.

Instances of this kind are comparatively rare. We introduce the following one from a recent lecture of Dr. Graves, reported in Renshaw's Journal for May 16th, 1835.

"The following case of *acute inflammation of the œsophagus* is particularly worthy of your attention, on account of the extreme rarity of the disease, and because its symptoms have, for that reason, been either erroneously or imperfectly described by authors.

My friend, Dr. Mackintosh, in his Elements of Pathology (vol. 1, p. 228), observes, 'That of all the structures in the human body, the œsophagus is perhaps the least liable to disease. In general it is difficult to detect inflammation of the œsophagus till ulceration and constriction take place. I have seen only one case of universal inflammation of this tube not caused by poison,' &c.

It is no wonder, therefore, that the description Dr. Mackintosh gives of the œsophagitis is very imperfect. The same may be said of that given by others. The best description of the disease is that given by J. P. Frank in his Epitome. If I recollect right, Abercrombie has recorded one well-marked case of œsophagitis. Strange enough, this disease is not spoken of at all in that excellent work, the Cyclopædia of Practical Medicine.

The inflammation in the following instance was evidently the result of cold, and occurring in a healthy habit, it ran through its course in a few days. The case is in the gentleman's own words, for when the disease was cured I requested him to give me a short account of it in writing.

'February 24th, 1835.—For some days I felt as if I had caught cold, with something like sore throat. I felt as if the root of the tongue at the left side was sore. By degrees this extended downwards; a ring about the lowest part of the throat became painful on swallowing. The pain was most sensible at the left side.

'26th.—I took a bit of bread before dinner, and, on attempting to swallow it, perceived great pain from the commencement of the throat, proceeding downwards towards the chest, as if the bread was then impeded by something, and from thence it seemed to proceed with increased pain to the back between the shoulders. I felt no want of appetite at dinner, but the attempt to swallow caused considerable pain. The night was passed in a state of great restlessness and with headach, violent pain sometimes seizing me on some little change of position, as it does in lumbago. The pain then seemed to affect the whole chest, and, extending to the back, caused a hot, burning sensation directly between the shoulders.

'27th.—On attempting to swallow, I felt such pain as to force me to cry out as if the entire passage from the throat to the stomach was inflamed, and that every thing, whether fluid or liquid, had to force its way painfully through the passage. In swallowing it seemed doubtful whether the food could proceed.'

So far the details were furnished by the patient himself. In addition I may remark that, on the 28th, the inflammation had evidently begun to diminish, and that in the course of a few days more it had entirely disappeared. The treatment was restricted to abstinence and antimonial diaphoretics. There was no redness to be seen in that part of the throat which is visible when the mouth is opened.

---

### Enteritis.

The following case is introduced in one of Dr. Graves' excellent lectures, published in our contemporary, the London Medical and Surgical Journal, (Renshaw's) for May 16th, 1835. It is characterised by the talented lecturer, as a case of "VIOLENT ENTERIC INFLAMMATION," communicated to him by his friend, Dr. Nolan. We shall state the case before we make any remark.

"On Monday evening, 27th February last, he casually complained of pains in his bowels; they had not been freed on that day, and supposing it an instance of mere indigestion, I ordered him five grains of calomel, and a draught of castor oil. For that night I heard no more of him, but, early on the following morning, I was hastily summoned by one of his fellow-servants, who reported that he was dying. I found him labouring under severe but intermitting pain of the belly, particularly about the umbilicus, *violent and frequent cramps*, especially of the lower extremities, and occasional vomiting. The surface was perfectly cold; features sunken; eyes surrounded by a dark areola; voice subdued to a whisper; pulse 140, small and feeble; abdomen tender though not at all tumid. He told me he passed the night in great torture, and that the bowels were still unmoved. I immediately ordered ten grains of calomel, to be followed in two hours by an oil and turpentine draught, a turpentine enema, bathing, &c.

Three hours subsequently—temperature restored; cramps less violent; vomiting less frequent, but bowels obstinate; face and pulse equally unpromising as before; abdominal pain increased. Was this incipient inflammation? and what is the cure for inflammation? Bleeding? Well, I did bleed; but scarcely had four ounces been taken, when I was very glad to tie up the arm; the prostration alarmed me. Something at all events ought to be done, and I ordered a sinapism to the abdomen, a repetition of the enema (for I confess I have not much confidence in frequent or powerful purgatives), a powder composed of calomel two grains, opium quarter of a grain, to be taken every fifteen minutes. Towards evening I thought my patient rallied a little; his countenance was better; pulse firmer; his abdominal pain not increased, and he vomited but once; the injection brought away with it a little mucus, but no more. Repetat haustus terebinth. Repetat quoque enema. During the night there was just a trace of feculent matter, but vomiting returned, and I found him in the morning (the second of his illness) suffering an increase of pain; the abdomen, too, was now not only extremely tender, but *decidedly swollen;* the pulse remained quick and weak as ever. I could not discover that he passed water. Would you not call this inflammation? But would you bleed for it? I did unfortunately to as great an extent as I could, which was about eight ounces, and the cadaverous look, the cold-clammy surface, in short, the absolute collapse which succeeded and *continued for hours*, gave me strong reason to regret it. *It produced no impression* upon the pain. I had read with great interest the invaluable observations of yourself and Dr. Stokes, as well as the publications of Armstrong, Griffin, Gooch, &c., wherein the applicability of opium to certain modifications of abdominal inflammation is forcibly demonstrated, and I thought my patient precisely in the condition in which you would probably have recourse to that powerful agent. I therefore commenced exhibiting half a grain of opium and two of calomel every half hour. After the second hour, I substituted for the calomel three grains of carbonate of ammonia, which, with the opium as before, I continued during the day and the whole night. In the morning (the third) I had the satisfaction of ascertaining that the pain and swelling had considerably subsided, and that the *bowels had been twice opened;* the countenance now spoke promisingly, and pulse began to fall. I however persevered in my plan of treatment for the day, and, indeed, for the two following nights and days (gradually increasing the interval between each dose however) until all trace of pain and obstruction had disappeared. The bowels continued to act from time to time, although I never ventured upon another purgative; the dejections were at first largely mixed with blood and mucus, but soon assumed every character of health. Of the sequel (may be the consequence) of this interesting case, you most kindly undertook the management, and I shall add nothing to this meagre statement of *facts*, which Mr. O'Donnell (of Keane's, in Suffolk-street), to whose humanity and care I am deeply indebted, witnessed as well as myself. I shall leave you to speculate upon the propriety of bleeding at all under such circumstances. I shall also leave you to decide whether the increase of inflammation, which certainly occurred when I first gave up the opium plan (on the first night) for the sake of interposing a purgative, was to be attributed to the change or not. May not the case throw some light upon the use or abuse of purgatives? But I am doing more than I intended, and more than is useful.

I remain, my dear Doctor,
Yours most truly,
J. NOLAN."

With every respect for the acumen and practical tact of Dr. Graves, we cannot bring ourselves to believe that there was one atom—we had almost said *element* of enteritis in the above case. If forced to give it a name, we would call it *enteralgia*, rather than *enteritis*. Whether or not it would have merged in inflammation of the bowels we cannot say—our impression is, that inflammation did not take place. Spasm may and does terminate—sometimes pass into inflammation; but, in the above case, we do not believe it had passed the boundary. Whatever the disease may be called, we have no hesitation in saying that Dr. Nolan's *treatment*, in our humble opinion, was the right one.

---

### PHYSICAL SIGNS *versus* ORDINARY SYMPTOMS—ORGANIC DISEASE OF THE HEART AND THE KIDNEYS.

The following very interesting case came lately under the observation of Dr. Johnson, and may be worthy of record, on account of some peculiar circumstances attending it.

A gentleman (Mr. W——), aged about 40, had been valetudinary for some years; but had attended to his professional duties, those of an architect, till within a month or six weeks of his death. Five weeks before this event, he walked, in one day, a distance of some ten or twelve miles. The most prominent symptoms of the complaint, after this, were total loss of appetite—the rejection from the stomach of almost every thing that was swallowed, whether food or medicine, and distressing sleeplessness. He had been seen by Dr. Hodgkin, and was daily attended by Mr. Rix. When Dr. J. first saw him, on the 30th March, Mr. Rix could not attend, and Dr. J. on examination, found the following symptoms:—Tongue furred with the thickest crust that could be imagined—great gastric irritability—signs of hypertrophy and dilatation of the heart to a considerable degree—chest sonorous throughout, except in the region of the heart—skin cool—urine apparently natural—no sleep—great prostration of strength—bowels uncertain—secretions unhealthy. Dr. J. wrote to Mr. Rix, that there was organic disease of the heart, and recommended effervescing salines, with small doses of Battley, in order to quiet the stomach, and procure some sleep. On Wednesday, the 1st April, Dr. J. and Mr. Rix met. The gastric irritability was considerably allayed, and the patient had got some sleep during both the preceding nights. Mr. Rix now pointed out to Dr. Johnson the state of Mr. Ward's urine, which was highly coagulable, both by heat, and when tested with nitric acid. The patient complained of no pain in the back or in the region of the kidneys, but there was tenderness, on deep pressure, in the situation of the right kidney. Although the disease was clearly of a fatal nature, yet, considering that Mr. W. had been able, two or three weeks previously, to walk several miles in one day—considering, also, that the lungs were sound, and the breathing quite unembarrassed, it was evident, both to Dr. J. and Mr. Rix, that the organic disease of the heart was not the sole cause of the rapid prostration of strength—the inability to retain any thing on the stomach, and the vigilium under which the patient laboured. An accurate examination of the epigastric region disclosed no symptom of inflammation in that situation, there being neither tenderness nor distention. The inference drawn by both practitioners then was, that, in addition to the organic disease of the heart, there was another disease—that of the kidneys, producing the irritability of the stomach by sympathy, and, in fact, being the main cause of the existing dangerous state of the patient. It was agreed that they should procced on the soothing plan, with saline medicines, alteratives, and small doses of Battley or morphine. Upon this plan, though the patient made no advance towards recovery, yet he passed the time more comfortably, and many of the symptoms—those of a sympathetic nature—were mitigated. The anxiety of friends brought Mr. Wray, of Salisbury-square, into consultation on Monday, the 6th April. Mr. Wray, without pronouncing any decided opinion as to the pathology of the disease, strongly urged the propriety of bloodletting, in small quantity. About three or four ounces were abstracted, when faintness occurred. The blood was a good deal inflamed; but no alteration appeared in the state of the patient, excepting progressive debility. The mineral acids were given, but without much sensible effect.

On Friday, 10th April, an eminent physician and a well-known author, was added to the consultation. Dr. C— had examined the patient the preceding day, in company with Mr. Rix, and had made up his mind as to the nature of the disease. His decided opinion was, that the disease was of an inflammatory nature, and seated in the epigastric region—namely, in the stomach or its immediate neighbourhood. He contended that depletion was the best plan of treatment—that opiates should be discontinued—and considered the case as by no means hopeless. No auscultation was practised, and Dr. C. could see no proof of organic disease either in the heart or kidneys. Dr. J. then wrote down on a slip of paper the two conflicting and opposite opinions of Dr. C. and himself, which he presented to Dr. C. and then deposited in the hands of Mr. Rix. As Dr. J. would not agree to farther bloodletting, it was deferred for that time, Dr. C. prognosticating that it would be necessary in a few days. In accordance with Dr. C.'s wish, the opiates were discontinued.

In an interview with the relations of the patient, it was rather imprudently stated by Dr. C. that Dr. Johnson entertained a much more gloomy opinion of the case than he (Dr. C.) did, as he (Dr. C.) considered it by no means hopeless. The result proved that this declaration made an unfavourable impression on the friends—for neither of the physicians were called in afterwards. The patient lingered a week longer, without taking any medicine, and then died.

Neither Dr. C. nor Dr. Johnson attended at the examination of the body, which was performed by Mr. Rix, in the presence of Dr. Hodgkin and Mr. Wray. The following memorandum was kindly furnished by Dr. Hodgkin, as copied verbatim from his note-book.

### Memorandum.

"Mr. G. Ward, aged about 40, of short stature and compact make, had led an active regular life, but was liable to exposure in the course of his profession, as master-builder. He had also suffered from mental distress, occasioned by the death of his wife. His health had been declining for some months; but serious apprehensions were not entertained until a few weeks before his death. He was seen once by Dr. Hodgkin, about three weeks or a month before his dissolution, when his peculiar sallow complexion, with slight fulness below the under eyelids, excited immediate suspicion that he was labouring under the renal disease, described by Dr. Bright, although no anasarca was observed in the legs or elsewhere. This gentleman complained of palpitation of the heart, unaccompanied by irregularity;—it was, therefore, considered to be enlarged, without valvular disease. Digestion was much impaired, and the odour of the breath was peculiar, and highly offensive. It may be observed that this gentleman's urine, when examined, proved to be highly albuminous—which character it retained, but, as Dr. H. was informed, to a still greater extent, till the last. It was of a pale colour, and not deficient in quantity. The evacuation of this fluid seemed almost to act like hemorrhage, in reducing the patient's strength.

*Autopsy.*—The head was not examined. The lungs were remarkably healthy, with very slight effusion of lymph on one side. There was a very small quantity of fluid in the pericardium. The heart was considerably enlarged. This change had principally taken place in the left ventricle, the parietes of which were above an inch and a half in thickness. The valves appeared nearly or quite healthy.

The abdominal viscera, on their peritoneal surface, presented a dusky appearance. The mucous membrane of the stomach and intestines appeared to be of a healthy texture; but there was a small increase of vascularity in the course of the small intestines. It was partial, and appeared to be the result of congestion—probably cadaveric. The spleen was small, and rather soft. The pancreas was also small, its figure being rather irregular, but its texture unaltered. The kidneys were small, and appeared shrunk. The tunica adiposa was somewhat indurated, apparently by an old inflammatory state of its cellular membrane. It adhered strongly to the proper capsule of the kidney, which easily separated from the gland. The surface of the kidney, thus exposed, was granular, affording a good specimen of far-advanced, mottled degeneration. Amongst the numerous white spots, several were of a partially opake appearance. A section through the kidney shewed that their glandular structure had wasted, the distinction between the cortical and tubular parts being nearly obliterated. The pelves of the kidneys contained a little turbid and apparently puriform mucus. Dr. H. remarked that, for some hours after the inspection, he carried about with him the peculiar offensive, penetrating, and pertinaciously adhering odour, which he had repeatedly noticed as attending the bodies of those who had died of this peculiar form of renal disease.

*Finsbury Circus,*
*29th April,* 1835."

The following note was received next day.

P.S. "I omitted to mention that the glands of Brunner, in the duodenum, were considerably developed. This being of a morbid character was at least equivocal, as it was not greater than often exists independently of disease. In order that I may not be considered to suppress an iota, I may add that there was some sanguineous discolouration of the mucous membrane of the stomach, but which I had no hesitation in regarding as rather cadaveric, or the result of events immediately preceding death."

J. H.

*30th April.*

The foregoing case is exceedingly interesting, as proving the insidious nature and fatal tendency of renal disease, where not a symptom was present, except the albuminous urine, that would have aroused suspicion in any mind, that such a malady was rapidly bringing life to a close. But then the albuminous urine was a physical sign which deserved the most attentive consideration, and which led Dr. Johnson, and, before Dr. J. saw the patient, which led Dr. Hodgkin to the very same conclusion. Dr. H. determined, from physical signs, in conjunction with general symptoms, that Mr. W. laboured under organic disease of the heart and kidneys. The disease of the heart, though of a formidable nature, and which would inevitably prove fatal in the end, was not advanced to that prominence, which would account for the rapid prostration of strength, and general break up of the constitution. The albuminous urine was, therefore, considered as of itself, in this case, sufficient evidence of renal disorganization. The extremely furred tongue was looked upon by the second consultant physician, as indicative of inflammatory disease in the stomach or its neighbourhood, especially when coupled with nausea and sickness. Now, independent of the proofs afforded by dissection, it may be safely inferred that a thickly-furred tongue is far less indicative of gastro-enteritis than it is of irritation, or a disorganizing process going on in some distant part, and affecting the stomach secondarily. A red tongue is infinitely more demonstrative of gastritis, or gastro-enteritis, than a white. In respect to the inflamed state of the blood, it is well known that, in hypertrophy of the heart, and in many other diseases, the buff will present itself till the last drop of blood is drained from the body. Look, for example, at pregnancy, where buffed blood affords no proof of inflammation.

A point of great importance in medical polity has been only slightly glanced at—the imprudence, to give it no worse a term, of letting any discrepancy among the consultants slip out among the friends, whilst delivering the opinion. In the foregoing case, the mischief was evident and irremediable. The friends naturally concluded that, where the physicians differed in *prognosis*, they would be likely to differ about the nature of the disease and the mode of treatment. The consequence was, that both were discharged—and that *one* must suffer in reputation by the event of the case. Is this discreet—is it just, that not merely the chance, but the certainty of injury should be incurred, by the voluntary disclosure to friends of discrepancy of opinion among the medical attendants? It is true that, in the present case, the injury fell on the party who unnecessarily incurred it. But this might not have been the case. The party who disclosed no discrepancy of opinion might have been the sufferer, and that for no other fault than an error of judgment—to which we are all liable, and for which no man should be subject to punishment. We do not believe that the consultant who made the disclosure had any intention or expectation of injuring his fellow-consultant; but a moment's consideration must have convinced him, that one or other must lose credit with the family in the end—and if he was confident in the truth of his own prognosis, he must have foreseen the danger that was impending over his colleague. There is one argument, however, which may be adduced in favour of the disclosure alluded to. The first consultant had given an unfavourable prognosis, and the second consultant not taking the same view of the nature of the disease, came to a different conclusion as to the final event, and might, therefore, deem it conscientious to disclose this discrepancy of opinion to the patient or friends. To this it may be answered, that *silence* as to the difference of professional opinion could do no injury to either party—whereas the disclosure must injure one of them. Are we, then, justified in injuring another for the sake of gaining some additional reputation ourselves? Suppose, for instance, that, in the present case, no allusion had been made to the discrepancy of opinion among the consultants. The recovery of the patient, contrary to the prognosis of the first consultant, could not possibly have injured the reputation of the second consultant—quite the reverse. The natural conclusion of the patient and friends would have been that, as Dr. Johnson was wrong in his prognosis, he was, most probably, wrong in his treatment of the case—ergo, the recovery of the patient was owing to the second consultant. Taking it in every point of view, then, we conceive that, unless upon some very extraordinary occasion, where great difference of opinion obtains as to treatment, there should be no disclosure of such opinions to the friends. If great discrepancy of opinion, upon some important point of treatment, occurred, we think it would be more prudent for one of the parties to retire, or to request a third opinion to decide the question.

In other respects, the case is extremely important. We have here an instance which proves the necessity of availing ourselves of physical and chemical signs, in addition to ordinary symptoms. It would probably be wrong to affirm that the physical signs are superior to the ordinary symptoms; but it would not be too much to say, that he who neglects to combine the two classes, in the investigation of diseases, does an injury to his patient and to himself in the long run. The case in question shews the necessity of examining the urine by other tests than litmus paper. The urine, in this case, appeared to the eye to be very natural, and the application of the litmus and turmeric tests, did not prove anything satisfactory. But heat and nitric acid immediately shewed that a serious disorder, if not disease, was going on in the kidneys.

P. S. This case occupied the London Medical Society during two sittings, the discussions being very animated. It was attempted to be maintained that the trifling and partial blush of redness in the mucous membrane of the stomach and small intestine was the product of inflammation, and justified the diagnosis of the second consultant physician!! We will not insult the understanding of our readers, nor expose the fallacy of the advocates of this view of the case, by deigning to answer it. It is equally false and ridiculous. A more interesting question was raised as to the cause of albuminous urine, and the effects of its discharge by the kidneys. It was considered by some experienced practical surgeons in the Society, that, in such cases, the kidneys were in a state that incapacitated them for the secretion of urine, and that the serum of the blood thus escaped, with destructive debility to the constitution.

---

### PATHOLOGICAL ANATOMY.

[*Concluded from page* 144.]

### Diseases of the Lungs and Thymus Gland.

Diseases affecting the tissue of the lung are incontestibly the most frequent to which newly-born children are subject. In many cases the disease appears to have existed for a few days only before birth, or even to have been the consequence of the commencement of the act of respiration. The partial development of the lungs, or pulmonary infiltration observed in the fœtus born before its time, and which breathes for several hours or even days, are evidently, in M. Cruveilhier's opinion, the result of the establishment of the function of respiration in lungs not yet adapted to it.

In the greater number of cases the disease has existed for several days or even months. The lungs then present different appearances. Sometimes they display the lobular pneumonia. Lobules, or groups of lobules, indurated or infiltrated, are dispersed in the midst of healthy tissue. Sometimes the lungs are extensively affected; M. Cruveilhier has seen both invaded throughout at once. The altered tissue sometimes resembles that of the pneumonia of the newly-born, at others it is completely carnified, granular, and the lobules represent glandular grains perfectly distinct. Lesion of the lungs is so frequent in the fœtus, that M. Cruveilhier has no hesitation in asserting that, *as many newly-born children die of disease of the lungs as adults.*

Such children die with asphyxia, but this differs from the asphyxia which is said by accoucheurs to be the cause of death, inasmuch as the latter is the mere result of the labour. The greater number of those who are said to die of such asphyxia do in reality die of apoplexy. The newly-born may live with a much less sound quantity of lung than the adult, and for reasons sufficiently obvious. Induration of the lungs may exist in the fœtus with embonpoint, and the appearance of perfect health. The bronchi are frequently filled with thick mucus, as in acute catarrh. The lungs of the fœtus may display much more extensive lesions than are sufficient to destroy the life of the adult.

M. Cruveilhier relates a considerable number of cases illustrative of the lesions alluded to.

A great number of children affected with syphilitic pustules have died of pneumonia or other lesion of the lungs, originating anterior to birth. Four cases are related. In the first there were cutaneous pustules, and the lungs were infiltrated here and there with blood and serum. In the second case the cutaneous pustules were connected with masses of induration in the lung, containing pus, and suppuration between the dura mater and frontal bone. In the third case the cutaneous pustules were accompanied with a greyish induration of the lungs throughout their whole extent; the spleen was much enlarged. In the fourth case the cutaneous pustules were accompanied with complete induration of both lungs.

M. Cruveilhier observes that, during the progress of gestation, the ovum may be considered a part of the mother, and is liable to disease as she is. It may be affected with pleurisy, pneumonia, peritonitis, chronic inflammation of the intestines, ascites, hydrothorax, syphilis, eruptions, even scirrhus. Of some of these affections he specifies cases for which we refer to the fasciculi themselves, or to 19th volume of this Journal.

### Diseases of the Mouth and Pharynx.

Figures 1, 2, 3, of the third plate of the fifteenth fasciculus are devoted to the delineation of aphthæ, le Muguet. M. Cruveilhier observes, that this affection is sometimes confined to the buccal membrane, sometimes extends to the mouth and pharynx, and sometimes attacks the œsophagus into which it penetrates more or less deeply. It is most commonly abruptly stopped at the termination of the epidermis of the œsophagus at the cardia. Under the false membranes that constitute the affection, and which are sometimes confluent, the mucous membrane is invested with its epidermis, and presents no other alteration than a redness, slight when the patches are distinct, intense when they are confluent. The muguet rarely penetrates into the air-tubes. Sometimes the circumference of the superior orifice of the larynx is studded with it, whilst the mucous membrane within is free. But M. Cruveilhier delineates one instance of implication of the ventricles of the larynx. The linear character of the pseudo-membranes of the œsophagus depends on the longitudinal folds of the mucous membrane.

M. Cruveilhier remarks that the muguet is nothing else than pseudo-membranous inflammation of the mucous membrane of the mouth. All ages are subject to it, though in the adult it is rarely idiopathic, but almost always symptomatic of some grave lesion of the intestinal canal, and appearing at a more or less advanced period of acute and chronic diseases, sometimes even during a false convalescence. It usually bodes a fatal termination, especially where it resists treatment. It may extend in the adult, as in the infant, to the pharynx and œsophagus, which our author has on several occasions found filled with a pultaceous whitish or brownish matter. In newly-born infants it is often epidemic, and it is endemic in certain hospitals where the wards are cold, humid, and ill-ventilated. This is the case with the hospital of Limoges, when the disease formerly committed great ravages. M. Cruveilhier doubts the advantage of the hospital system for the newly-born. The milk of a good nurse and pure air are much more advantageous than medicine.

### Follicular Ulceration of the Stomach.

M. Cruveilhier remarks that this form of ulceration has been described by M. Belliard, who has published fifteen cases. In eight of these the children were from four to six days old; six were from eight to twelve; one was aged three weeks; proving, so far as a few facts can do so, that the nearer birth, the greater the prevalence of this affection. Several of the patients had severe lesions in other organs; one only, aged four days, had no other lesion than ulceration of the stomach.

Figures 4, 5, 6, of plate III. represent this lesion in the stomachs of children who died, one on the eighth day, another on the fifteenth, and the third one month after birth. In all, the ulcers were more or less numerous, and some had run into each other.

### Diseases of the Spinal Marrow.

1. *Spina Bifida.* M. Cruveilhier presents the sum of the particulars of seven cases of spina bifida. Five occurred in infants born at the Maternité during the time he was physician to that hospital. The history of all is nearly similar, all being otherwise well formed at birth, and enjoying free motion, even in the lower limbs, with the exception of one child in whom they were semi-paralysed.

Out of the five cases that occurred at the Maternité, the tumor was perforated at birth in two. M. Cruveilhier thinks that the perforation occurred at the moment of delivery, it appearing to be recent, and the liquid that escaped being perfectly limpid. In these two cases, the tumor was much larger than in the three in which no opening existed. As the thinness of the parietes of such tumors is directly as their size, the larger they are the more likely are they to be ruptured in delivery. M. Cruveilhier appears to entertain no doubt of the possibility of an ulcerated opening occurring before birth, for ulceration and commencing cicatrization are not unfrequently seen upon the surface of the tumor.

In the whole of the cases, the tumor occupied the space comprised between the first lumbar vertebra and coccyx, the most common seat of spina bifida, which, however, has been seen in the whole length of the vertebral column. The greater frequency of its occurrence in the lumbar region is satisfactorily explained by the successive development of the spinal arch and laminæ from above downwards.

The constant co-existence of spina bifida and hydro-rachis has made them bear the general relation of cause and effect, the accumulation of fluid occasioning the separation of the vertebral laminæ. The analogous instance of hydrocephalus bears out this reasoning. But M. Cruveilhier thinks that, unless the spinal canal were peculiarly circumstanced, the mere accumulation of fluid, would produce distention only of the membranes, not hernia of them and of the spinal bones. After asking his readers and himself two questions,—first, whether those peculiar circumstances consist in a deficiency of continuity of the osseous canal, and of interruptions between the vertebræ? and, whether the spinal membranes distended by the fluid at first protrude into those inter-vertebral intervals, and subsequently, by the distention and pressure which the accumulating fluid exercises, separate the laminæ to the right and left? After asking these questions, we say, M. Cruveilhier declares that he is convinced, by the dissections he has made, of the impossibility of accounting for these tumors, in any other way than by admitting that, prior to the formation of the cartilaginous laminæ, the medulla and its envelopes have contracted an adhesion to the skin, an adhesion which keeps the medulla out of the vertebral canal, and consequently opposes the formation of the laminæ in this situation. This explanation he thinks sufficient, and indeed indispensable for the solution of the phenomena.

The connexion between hydrocephalus and spina bifida has been frequently remarked. In the majority of cases which have presented themselves to our author, he has found a certain quantity of serum in the cerebral ventricles. In one case, the ventricles were not unusually dilated, but the sub-arachnoid cellular tissue was filled with serum; and in two other cases, the brain was in a healthy state.

M. Cruveilhier's dissections enable him to confirm the accuracy of those observers, Tulpius, Burgius, Morgagni, &c., who have pointed out a particular disposition of the nerves and medulla, as common in cases of spina bifida. From a very careful examination of four cases, M. Cruveilhier ascertained that, in all, the spinal marrow with its membranes lost itself in the parietes of the tumor, and that from this part of the medulla, sometimes sound and sometimes atrophied or softened, sprang the nerves, which occasionally are even larger than natural. Another circumstance which has struck M. Cruveilhier, is the fact that, in spina bifida occupying the sacral region, it is not the cauda equina nor the sacral nerves which are confounded with the parietes of the tumor, but the spinal medulla itself. The explanation of this must be looked for in the condition of the medulla in the fœtus, in which it occupies the entire length of the vertebro-sacral canal.

The adhesion of the spinal marrow to the tumor, and the alteration that its inferior extremity undergoes, account for the weakness of the lower limbs, and even paraplegia, under which some infants suffer.

The symptoms are briefly specified by our author. The tumor, in the cases that have fallen under his observation, was fluctuating and semi-transparent—when gently and gradually compressed, the infant uttered no expression of pain, but it cried if the pressure was rudely made. The tumor was evidently distended by the act of respiration, and by crying, and once M. Cruveilhier perceived it pulsate gently in correspondence with the pulse.

So long as the tumor remains unopened, the infant is generally well in health, but when opened, the child is generally, after the lapse of a few hours or days, attacked with fever and convulsions, succeeded by paraplegia and death.

On cadaveric inspection, pus, sometimes of a serous character, sometimes pseudo-membranous, is found in the sub-arachnoid cellular tissue, and this may be the case to the extent of even forcibly distending the dura mater. In other instances, when death has rapidly occurred, the spinal vessels are found much injected, and the sub-arachnoid cellular tissue is infiltrated with opaque serum, but not with pus. The inflammation commonly extends to the sub-arachnoid cellular tissue at the basis of the brain, and to the lining membrane of the ventricles.

M. Cruveilhier has constantly observed, at the level of the first cervical vertebra, a sort of elongated pouch, filled with serum or with pus, and formed out of the spinal arachnoid. This must exercise some pressure on the medulla oblongata. He has found the spinal marrow sound in all cases but one, where its inferior part was softened for the space of several lines, and contained, in the centre of the softened part, a clot about the size of the head of a pin. In several instances the pus has occupied both the cavity of the spinal and cranial arachnoid, and the sub-arachnoid cellular tissue.

M. Cruveilhier denounces puncture of the tumor with any instrument, as a means of cure. Sir A. Cooper employed in one instance frequent punctures, and the case did well. But this is looked on by M. Cruveilhier as an escape rather than a cure. The seton and the ligature, he thinks, and with justice, are fraught with danger. The only rational method of treatment is moderate compression.

M. Cruveilhier concludes by impressively stating that spina bifida is not of itself a mortal malady, but that it becomes so only in consequence of the opening of the tumor, and the entrance of air into the serous cavity, an entrance almost inevitably succeeded by arachnitis.

2. *Case of Monstrosity.* The child which was the subject of the plates and notice of M. Cruveilhier was born alive, but died in five minutes after birth. It offered a remarkable example of displacements occurring simultaneously in the three splanchnic cavities.

1. There was hernia in the occipital and anterior cervical regions, the hernia being formed by the meninges protruded by a liquid.

2. There was hernia of the lung into the cervical region.

3. There was displacement of the small intestines, of a portion of the large intestine, and of two portions of the liver. The small intestine, the large intestine, and a portion of the liver occupied the left cavity of the pleura; while another portion of the liver occupied the right cavity of the pleura.

4. There was mediastinal hernia, the stomach, and the duodenum occupying the posterior mediastinum.

5. And, finally, there was invagination of the œsophagus within itself.

The fact is more curious than M. Cruveilhier's speculations on it, and, as both are of little practical utility, we dismiss them without any farther notice.

### DISEASES OF THE PLACENTA.

We may not unnaturally glance at these in connexion with the diseases that affect the fœtus. The knowledge of the alterations that this important part may undergo is not unworthy of the attention of practitioners.

The placenta, the physiological lungs of the fœtus, serves at once for the purification of the blood and the transmission of the nutritive material to the embryo. It consequently exercises a great and a double influence upon the latter. M. Cruveilhier states that the diseases of the fœtus exercise only a moderate influence on its nutrition, and that the operation of the diseases of the mother is inferior, in this respect, to that of the diseases of the placenta.

The alterations of the placenta are not numerous, and have been imperfectly delineated and observed. M. Brachet de Lyon has published ten cases of placental disease, with the view of establishing the relation that exists between them, the state of the fœtus, and maternal symptoms. He refers the placental alterations to inflammation, and describes the following effects of that process :—1, Engorgement of the placenta, which he compares to hepatization of the lungs; 2, A "scirrhous state," the result of chronic inflammation;* 3, Suppuration of the placenta; 4, Tubercular degeneration, occasioned by suppuration of several portions of the placenta; 5, Organic adhesion of the placenta and the uterus. Such is the arrangement of M. Brachet, an arrangement evidently squared and planed by the hand of a systematist.

M. Cruveilhier, thinks that the diseases of the placenta may be fairly reduced to the following heads.

1. *Hypertrophy.* This sometimes consists in a serous infiltration, analogous to what is so frequently observed in the umbilical cord. This was connected with a deposition of false membrane in the following case :—A woman miscarried at eight months of a dead child. The enormous placenta weighed a pound and three-quarters. It was infiltrated with serum, which pursued the direction of the vessels, and gave the placenta a considerable volume. The cellular tissue external to the chorion was filled with coagulable lymph, and that between the chorion and amnios was infiltrated.

2. *Atrophy.* This may be general, or only partial, and confined to some of the cotyledons. The consequence may be wasting, or even death of the embryo. The characters of placental atrophy are a shrinking, and a sort of desiccation of the placenta which becomes dense, often granular, and even tubercular in appearance, and of a yellowish-white colour. This alteration has been described by authors under the name of *scirrhous, tubercular placenta.* M. Cruveilhier looks on it as the consequence of a separation of the placenta from the uterus, at the points at which the atrophy takes place.

3. *Inflammation of the Placenta.* Independently of less decisive evidence, inflammatory affections of the placenta are proved by two of the cases detailed by M. Brachet. In one, the placenta was very large, and three-quarters of it were occupied by a large depôt of purulent matter, mixed with blood. This was seated on the surface of the placenta, which it separated from its membranes, whilst granulations were noticed on the latter. In the second case, M. Brachet found, on cutting into the placenta, a number of points, presenting all the characters of the red hepatization, tending to pass into the grey. Besides these, there were numerous small collections of matter, varying in size, from that of a pea to that of a filbert. The pus was thick, and not offensive, the cavity containing it was not encysted, and the matter seemed simply deposited in the structure of the organ.

* It is singular that in France, the land of pathological anatomy, the pathological condition of scirrhus should be so ill understood in general. The mass of French writers call all chronic indurations scirrhous.—EDS.

4. *Ossification of the Placenta.* There are varieties of ossification of this organ. In one, there is an osseous shell, of one, two, or three lines in thickness, covering uniformly, or in large patches, the uterine surface of the placenta, without penetrating into its substance. In the other, there are osseous spiculæ, which penetrate the organ, and traverse it in all directions. This kind of ossification proceeds always from the uterine to the fœtal surface. In a case related by Dr. Garin, each cotyledon had its osseous plate, and all the plates were bound together by an elastic fibrous tissue. The woman was confined at her full time of a living child. In a case related by M. Carestia, two concretions existed on the fœtal surface of the placenta, and a great number on its uterine aspect.

5. *Hydatid Cysts of the Placenta.* These are the most frequent of the morbid appearances in the placenta. They are serous, and not true hydatid cysts.

6. *Apoplexy of the Placenta.* We sometimes meet, in the torn substance of the placenta, collections of blood, varying in number and in age. Occasionally these are limited to a few, or to a single cotyledon; but often a greater number are affected, and then abortion is inevitable. M. Cruveilhier has met, in the same placenta, all the changes which apoplectic cysts undergo.

Here our present notice of M. Cruveilhier's labours terminates. We shall take care to bring the whole before our readers. For none can now neglect pathology, and a knowledge of its facts is indispensable, even to the humblest practitioner. Its advantages are not immediate, but it forms the foundation of all genuine medical and surgical science, and without it that science is false and fleeting.

---

### On the Management of Gonorrhœa.

This troublesome disease is sufficiently common, to make a knowledge of the best and most successful treatment an object of no inconsiderable importance. One would also be inclined to think that it is so common as to furnish to all the means of observation, and to enable a consistent and correct opinion of its nature and management to be formed. Unfortunately, however, this is not the case, and the reasonable expectation is not realized in practice. For opinions the most opposite and contradictory are entertained upon both points, and, as all parties boldly appeal to facts, the prospect of agreement, and the probability of settling the question by a satisfactory decision, would seem to be equally remote.

The identity of syphilis and gonorrhœa has, as our readers are well aware, been often affirmed, and as often denied. The proofs are hypothetical, the facts opposed to them are positive. The leading circumstance against the identity or similarity of the two poisons is the general tendency to the supervention of secondary symptoms in one, and the still more general indisposition to the occurrence of such symptoms in the other. This is a circumstance which must weigh more powerfully with a plain and a practical man, than all the specious arguments and pretty theories of ingenious reasoners, or than even the most imposing statements of actual experiments. It has been said, indeed, that secondary symptoms do follow gonorrhœa. Setting aside what is termed gonorrhœal rheumatism, and gonorrhœal ophthalmia, we must say we have never witnessed an unequivocal example of secondary symptoms after this complaint. Yet we have seen much both of syphilis and gonorrhœa; and we leave it to the theorists to explain why, the poison being similar in kind, phenomena so opposite should be displayed.

It is now a considerable time since we reviewed a work on the venereal disease, from the pen of Mr. Wallace of Dublin. Some portions of that work remain unnoticed, and amongst those portions is the section devoted to the consideration of gonorrhœa. We are anxious to bring the subject of venereal maladies frequently before our readers, because there is more ignorance with respect to their true character and proper treatment than could be wished. We shall, therefore, select the present chapter of the work of Mr. Wallace for notice and for criticism.

Mr. Wallace displays throughout this work a peculiar tendency to theorize. With that lubricity which is said to characterise the eel of science, he slips from position to position, and from fact to fact, with a dexterity more calculated to give birth to admiration than conviction. Admit but his postulate, and you are overwhelmed by the sequiturs.

Gonorrhœa is with him a kind of syphilis, and, oddly enough, he places it as an intermediate link between phagedæna and indurated syphilis, or what is vulgarly considered the beau-ideal of a chancre. Our readers may stare, as all plain men would, at such a localization, yet such it receives from Mr. Wallace, and a long and curious process of reasoning is employed to establish its propriety. We may disregard all this, and, picking the insulated gem from the cabinet, proceed to inspect and to admire it.

Gonorrhœa, according to Mr. Wallace, is analogous to erysipelas, as regular syphilis is similar to phlegmon. As an illustration of the useless ingenuity that likens things unlike, we may quote the passage that contains the parallel.

"It has always seemed to me, that catarrhal primary syphilis holds, in some measure, the same relation to that form of inflammation called erysipelas, or erratic inflammation, or diffused inflammation, as the regular disease bears to the phlegmonoid or circumscribed form of inflammatory action. For the regular form of syphilis is characterized by its morbid action being circumscribed like that of phlegmon; while the catarrhal form is equally characterized by a disposition to be diffused, to creep from one part to another, &c., like erysipelas. In fact, the morbid action in catarrhal primary syphilis may so extend as to involve, at the same time, the whole of the urethra, the bladder, the testicles, the glans and prepuce, in the male; and in the female the nymphæ, labiæ, clitoris, vagina, &c. &c.: while its tendency to creep from one part to another—decreasing or disappearing in a great measure in the parts first affected, but extending in the same proportion to the sound parts—is oftentimes so great, that in the male, in whom it is generally confined at its origin to the preputial end of the penis, it not unfrequently creeps slowly on to the posterior parts of the urethra, to the bladder, or to the testicles, while it decreases or ceases entirely in the parts first affected. Hence we sometimes find, that these more deeply-seated organs are severely affected after the disease has ceased, or nearly ceased, in its original seat. Thus also the discharge may be often forced, by pressure made along the penis, to come from parts of the urethra more or less removed from its orifice, according to the stage of the disease.

The disposition to creep along a continuous surface, from one part to another, is not to be confounded with a disposition equally possessed by catarrhal primary syphilis in common with the erysipelatous form of inflammation, to excite disease in remote parts, without influencing perceptibly the intermediate parts. Of this mode of influence, we remark the following varieties:—

1st. The original disease may diminish or cease, immediately before the remote organ becomes affected; or else the remote organ may become affected, and then the original disease quickly decrease or disappear.

2d. The original disease, in place of disappearing or decreasing upon the occurrence of the secondary disease, may, by the reaction of the latter, be increased.

3d. The original disease may remain uninfluenced by the secondary disease.

When catarrhal primary syphilis produces sympathetic disease in remote parts, without influencing perceptibly the intermediate parts, I am not disposed, from the facts which I have observed connected with such occurrences, to consider the sympathetic disease syphilitic; for it seems to me, for several reasons, that the specific action of syphilis cannot be produced in any part, unless by the direct contact of venereal matter. I therefore consider such sympathetic affections to be forms of erysipelatous, or diffused, or erratic inflammation, excited by a syphilitic disease, although they are not themselves syphilitic."

The utilitarian in science may remark, that the comparison of gonorrhœa with erysipelas, and of syphilis with phlegmon, is absurd, if meant as a comparison of diseases, and useless if intended as a comparison of actions. It savours, in fact, of the old and idle mania for erecting processes, and actions, and stimuli into substantive things, for substituting the chimeras of a prurient imagination for the sober observation of facts. We defy the student, be he young or old, to rise from such comparisons as this of Mr. Wallace, with one more definite idea of the nature and the laws of gonorrhœa. On the contrary, he is confused by the mixture of fact and supposition, of actual circumstance and far-fetched analogy.

We do not know why Mr. Wallace denies the power of syphilis to contaminate parts remote from that which was primarily affected, unless it influence, in a perceptible degree, the parts which are intermediate. When a man contracts a sore upon the penis, and, after a period of five or six weeks, exhibits an ulcer in the throat, we generally believe that the latter is a secondary syphilitic symptom, although no intermediate part is implicated. How Mr. Wallace may explain this difficulty, we cannot, of course, pretend to determine; but no doubt his ingenuity *can* explain it.

Mr. Wallace's views of the nature of gonorrhœa are, as Bentley said in amending a celebrated passage in Milton*—

"Not light, but rather a transparent gloom."

"The very uncertain effects of remedies employed in the treatment of catarrhal primary syphilis, and the numerous and remarkable phenomena which it often presents during its progress, seem to prove that it is a disease of a very complex character. But do we consider with sufficient accuracy the nature of the morbid actions which constitute this disease? And is not our treatment of it more empirical than it need be, in the present state of our knowledge?

That catarrhal primary syphilis is not, as some appear to believe, a purely inflammatory disease, must be admitted, when we consider the effects which it sometimes produces in the system, and that it cannot be subdued with certainty or rapidity by those measures which quickly cause the termination of common inflammation; yet its phenomena leave no room to doubt, that it is, at least in general, accompanied by a state of inflammatory action.

It might, from reflecting on the cause and occasional effects of catarrhal primary syphilis, be supposed probable, that the specific or peculiar mode of action, which is evidently superadded in this disease to the state of inflammation, is similar to that which attends the ulcerating forms of primary syphilis; but when we examine this question more closely, we must conclude that they are different: for, otherwise, we cannot account for the peculiar local characters of catarrhal syphilis, nor why mercury, which so often acts as a powerful resolvent, as it were, on the ulcerating forms of syphilis, should exercise an influence comparatively trifling on the catarrhal form. At the same time it must be admitted, that the morbid state which attends the latter partakes somewhat—perhaps sometimes more and sometimes less—of the mode of action of the former; for we know that they are capable of reciprocally producing each other, and of causing analogous secondary effects in the constitution.

Thus it is evident, that, as the phenomena of catarrhal primary syphilis are neither purely inflammatory nor purely syphilitic, there must exist a peculiar mode of action resulting either

* "Not light, but rather darkness visible."

from a combination of inflammatory and syphilitic action, or from the addition of an entirely new mode of action; and it is most probable that the latter is the case, as otherwise we shall not be well able to explain the peculiarities of this disease;—for although inflammation often complicates the ulcerating forms of syphilis, these forms never present, when this combination occurs, the characters of catarrhal syphilis.

In relation to this question we are, however, to remember, that the inflammation of catarrhal primary syphilis is more purely erysipelatous; and that a morbid state might result from the combination of this form of inflammation with the syphilitic action, which would not result from its combination with a more phlegmonoid or ulcerating form.

Now do not the preceding reflections authorize us to conclude, that the morbid state which constitutes catarrhal primary syphilis, is a complex one, formed of inflammation, of an erysipelatous character,—of a specific mode of action, which establishes a degree of relationship between it and the ulcerating forms of the venereal disease,—and, lastly, of a mode of action which is its particular attribute, or by which it differs from all other diseases? And is it not in accordance with the present state of our practical knowledge to admit, that the inflammation may subside, or be removed, while the syphilitic action remains combined with the specific action of the disease; or that one of the latter modes of action may persist, after the other has subsided or been removed?"

Well may Mr. Wallace designate this view as "somewhat hypothetical." It is a very pretty specimen of that form of reasoning known in the Green Isle under the name of botheration. Gonorrhœa is a combination of an inflammation, which is not an inflammation, but more an erysipelas—of a syphilis which is not a syphilis, but something else, a relation to it—and, lastly, of a mode of action which is its particular attribute, or by which it differs from every thing else. We make Mr. Wallace a present of his theory, and we make our readers a present of the meaning of it, if any meaning, stripped of verbiage, there be. And all this windy dissertation is substituted for a simple and straight-forward description of the phenomena of gonorrhœa—all this speculation, fanciful, and subtle, and idle speculation, for a simple consideration of its facts. Why, what treason have we here against the very essence of inductive reasoning, against the very spirit of the philosophy which Bacon taught, and which has made every science what it is. We think we see again the old array of dogmatists and empirics in medicine before us, we think we have the work of a writer of the last century, a writer of the wordy school of Hunter.*

When we turn from such fancies, engendered in the closet, to the simple observation of nature, we find the following circumstances. First, that gonorrhœa—that is, a discharge from the urethra, varying in colour and consistence, attended with more or less of inflammatory action in the mucous membrane of the urethra, and following sexual intercourse—is extremely frequent—secondly, that, in some cases, there is much of common inflammatory action, not only in the mucous membrane, but in contiguous tissues, whilst, in other instances, the inflammation is comparatively slight—thirdly, that parts connected, by continuity of texture, with the mucous membrane of the urethra, occasionally become affected with inflammation, the primary complaint ceasing, diminishing, or remaining in statu quo—fourthly, that the mucous membrane of the conjunctiva and the synovial membranes of the joints, are occasionally attacked with inflammation, as a consequence of the primary complaint—and, fifthly, that secondary symptoms are so rare, as to render their nature dubious and their cause uncertain. Such are the conclu-

* We suppose there are few, now-a-days, who will defend the mode of reasoning adopted by John Hunter. His great merits are to be found in his museum—in his almost superhuman labours.

sions, simple, and, we believe unbiassed, that our means of observation will enable us to form. Perhaps it would be well to institute an elaborate series of experiments on the communicability of gonorrhœa, and on the possibility of producing sores of a syphilitic character, and followed by such symptoms as succeed to syphilis, by inoculation of ulcerated surfaces with gonorrhœal matter. But difficulties stand in the way of such experiments, which can only be conducted with satisfaction and success in a public hospital.

We may proceed to discuss the treatment of this form of venereal complaint, a form more troublesome, in many instances, than those more formidable in their existing characters and secondary consequences. There can be no question that gonorrhœa has been treated too empirically—that surgeons have been led away from the observation of varieties of symptoms, and the application of remedies to those varieties, in order to discover one specific for all—that the presence or absence of common inflammation has been too much disregarded—and, as a natural result of such neglect, that stimulating medicines have been grievously abused. One man has lauded cubebs, another copaiba, a third the turpentines, a fourth injections, as a cure for gonorrhœa; and cubebs, copaiba, turpentine, injections have been as blindly given as indiscriminately recommended. The result has been, an extreme and unnecessary aggravation of inflammatory symptoms and inflammatory complications. Inflammation of the prostate, of the bladder, of the testis, induration of the corpus spongiosum, enlargement of the lacunæ, and stricture, have been as frequently the fault of the surgeon as the natural consequence of the disease.

In some former Numbers of this Journal, we pointed out the principal inflammatory symptoms attending gonorrhœa, and endeavoured to lay down some rules of treatment which might tend to diminish the obstinacy of the complaint, and to obviate the unpleasant accidents that not unfrequently attend it. It was chiefly with the view of following up the spirit and the matter of those remarks that we began this article.

To the treatment of gonorrhœa we shall, therefore, proceed, making Mr. Wallace's text the peg on which to hang any observations of our own. He directs attention, and properly directs it, to the existence or the absence of inflammatory symptoms—and to the habits and constitution of the patient. The surgeon should make himself accurately acquainted with the duration of the symptoms, the treatment that has been adopted, and the history of former attacks of gonorrhœa, if any such have been experienced.

It is generally thought that a first gonorrhœa is the worst, and so it frequently is. But occasionally this is not the case. In January, 1833, we were consulted by a patient, who was then labouring under gonorrhœa, with severe inflammatory symptoms. It was perfectly cured in one month. This was the fifth attack of the complaint. The first had occurred seven years previously, and lasted from three to four months—the second, twelve months after the first, and had lasted from six to seven months—the third twelve months after the second, from four to five months—the fourth twelve months after the third, from nine to ten months. We might mention many instances of this description, but every practical surgeon must have met with such.

On the symptoms indicative of inflammatory action, we think we need not dwell—they are obvious and familiar. But we may make a remark or two on the discharge. During the inflammatory stage, it is usually yellow and thick. Mr. Wallace remarks, in reference to this point—

"It would seem as if there existed a close relation between the characters of the discharge and the state and degree of the attending inflammation. During the first stage, or before inflammation has commenced, and during the third stage, or when it has become decidedly passive or has ceased, the discharge is, in general, more scanty, more thin, and more colourless, than during the intermediate stage, or when the state of inflammation is at its highest degree. I have also observed, that the less the inflammatory action, the less alkaline the discharge, as may be easily ascertained by litmus paper. When the inflammation is at its height, the discharge has very often a greenish tinge,—sometimes it is tinged with blood, and at others it is of a primrose yellow colour. These varieties in the shade of colour point out corresponding varieties in the degree and character of the inflammation. The thick primrose-coloured discharge generally occurs in more healthy habits, and the greenish and reddish discharges in weaker and more irritable persons. When the inflammation is very high, we may sometimes remark shreds of lymph discharged from the urethra with the urine."

We are disposed to agree with Mr. Wallace in the position, that a thick and yellow discharge is usually the sign of a sthenic habit and of sthenic action. But we do not coincide so entirely in the remark, that the reddish discharges are evidences of weakness. We have seen thick dark discharge, the colour being evidently due to the presence of small quantities of blood, in persons of a plethoric habit and of strong constitutions. We should say that we have found such discharges generally peculiarly obstinate. We have under our care at this moment a gentleman in whom this dark discharge has existed, and in whom the disease has proved peculiarly intractable. Capivi, cubebs, turpentine have arrested it, but each has done so for a few days only.

In some persons of an irritable habit, very great pain in micturition attends a thin and profuse discharge. Here there is inflammation, but probably not of a very acute, at all events not of a very healthy kind.

Are pain and chordee decisive evidence of inflammation? Let us hear what Mr. Wallace has to say.

"It might be presumed, that the state of sensibility of the affected parts would afford a satisfactory criterion of the state of inflammation. This, however, we do not find to be the case; for although the sensibility of the diseased surface decreases in general very much, as soon as the atonic stage of catarrhal syphilis commences, there often exists with asthenic action very considerable pain or irritability. We cannot, therefore presume that the active stage of inflammation still continues, merely because there may exist much *ardor urinæ*. But if there be a constant uneasiness, rather than an excess of sensibility to the passing of the urine, and more particularly if this constant uneasiness exists along with a recent disease, we shall, in general, be justified in concluding that a state of active inflammation is then present.

Are we to consider the accompanying inflammation sthenic, as long as chordee persists? Certainly not; for although chordee is very frequently connected with active inflammation, it is also often connected with an irritable and atonic state of the urethra."

We observe that Mr. Wallace takes no notice of a distinction which is practically important, a distinction between painful erections and chordee. Patients constantly say they have chordee, when we find on inquiry that they have pain in erection at the end of the penis, or in the course of the urethra, unattended with any actual curvature of the former. Now it must be evident from the least consideration of the structure of the urethra that true chordee, that is curvature of the penis in erection, cannot possibly occur without a physical alteration of the corpus spongiosum or corpus cavernosum of the penis. And such an alteration is and must be the effect of inflammation. But mere painful erections, though generally also the result of inflammation, are sometimes attended with comparatively little, so little that it may be almost disregarded. We are careful then to inquire particularly of patients the character of what they term chordee, and four times out of five, we ascertain that they have not really chordee at all.

On taking a review of the symptoms we have noticed, we may state in a broad and general way, that if a patient of a tolerably good habit of body, has purulent or even thin profuse discharge, and considerable pain in micturition, that patient has the inflammatory symptoms of gonorrhœa, and requires more or less antiphlogistic treatment. If the orifice of the urethra is red and pouting, if painful erections are experienced, and if any general pyrexia is present, the inflammatory character is still more marked.

The inflammatory complications of gonorrhœa, consist of affections of other tissues besides the mere mucous membrane of the urethra. They are inflammation, and induration or suppuration of the lacunæ—inflammation of the corpus spongiosum, or the corpora cavernosa—inflammation of the cellular tissue of the penis, producing phymosis, paraphymosis, or abscess—inflammation of the absorbents of the penis—inflammation of the prostate, the bladder, and the testis. Such are the occasional, and the unequivocal inflammatory complications of gonorrhœa. That they are inflammatory, is too obvious to require argument or proof. Yet strange to say, some at least, of these affections, have been disregarded, and their purely inflammatory character has been overlooked. On some of these complications or results of gonorrhœa, we have fully dwelt in former numbers of this Journal. What remarks we have to offer will therefore be brief, and will be better introduced farther on. Let us follow Mr. Wallace as he passes to the treatment.

During the inflammatory stage, that is, while there are genuine inflammatory symptoms, antiphlogistic measures are required. General bleeding is seldom necessary, but leeches to the penis or the perinæum often are so. On the other items of the antiphlogistic code we may be silent. Mr. Wallace mentions, among other remedies, mild laxatives, and opium and henbane with calomel. We must say we always give calomel with antimony and opium at first, and conjoin with these medicines not merely mild laxatives, but powerful cathartics. We are certain, quite certain that the latter are productive of very great service. What shall we say to the following heresy?

"Is mercury" says Mr. Wallace, "to be employed in catarrhal primary syphilis? It is my general practice to administer mercury in small doses in this disease, when it is not accompanied by a state of active inflammation, or when the active inflammation has been subdued; and this practice I have adopted upon the following grounds.

1st. The power possessed by the catarrhal form of primary syphilis to produce the ulcerating forms of this disease, points out a similarity in their morbid action, and affords some reason to presume that they may be combated by analogous measures.

2d. As the catarrhal form of primary syphilis may produce constitutional symptoms similar to those which follow the ulcerating forms of this disease, and as mercury is admitted to have a controlling influence in preventing those of the latter, it may be presumed that it will have a similar influence over those of the former.

3d. As mercury has a remarkable power over the catarrhal form of secondary syphilis, or that form of syphilitic catarrh which results from contamination of the system, we may, from analogy, conclude that it is likely to have some power over the catarrhal form of primary syphilis.

4th. If cases of catarrhal primary syphilis occur, upon which mercury does not seem to exercise any control, there occur other cases in which we find it to act in the most salutary manner; and others again, in which the discharge will continue, and be after a time followed by induration and bubo, and most probably by secondary symptoms, unless this medicine be given.

5th. If mercury does not serve any useful purpose in the treatment of catarrhal syphilis, it can do no harm if properly administered, nor will it interfere with the administration of any other remedies.

Small doses of mercury are sufficient in ordinary cases of catarrhal syphilis. I seldom employ more than about five grains of the blue pill with a quarter of a grain of opium twice a day, or morning and night; and seldom continue it longer than two or three weeks. This has appeared to me sufficient to afford all the assistance required in general for the removal of this disease, or to protect the constitution from contamination."

The majority of our readers, and the practical portion too, will feel some surprize at a writer recommending at this time of day, a *course* of mercury for gonorrhœa. The common sense of surgeons would seem to have settled this matter for ever, would appear to have decided that such treatment is at once unnecessary and injurious. For observe that this is not a new system plausibly and freshly advocated, but an old and abandoned one resuscitated. It is but a few years since Sir Astley Cooper denounced as infamous the antiquated custom then maintained in the Borough Hospitals, of putting gonorrhœal patients through a course of mercury.

Mr. Wallace is in general peculiarly unfortunate in his methods of reasoning. His principal argument is the petitio principii. Such is certainly the case in the present instance; such is the character of the assertion, for it is no more, that gonorrhœa possesses the power of producing the ulcerating forms of the disease. He does not even attempt to prove this; and until he does so, his readers must deny it. The second assertion is equally gratuitous:—that gonorrhœa may occasion secondary symptoms similar to those of syphilis. Of this he offers not a shadow of a proof, and the common experience of mankind is opposed to it. How often do we witness gonorrhœa: how seldom do we see any secondary symptoms from it. For our own parts, we repeat, what we have already stated, that we never yet saw one unequivocal instance of the sort. What "the catarrhal form of secondary syphilis, or that form of syphilitic catarrh which results from contamination of the system," may be, we profess ourselves perfectly unconscious. We therefore make Mr. Wallace a present of all the value, if any there is, of his third argument or statement, whichever it may prove. The fourth is a combination of two main assertions: first that mercury is in some cases "most salutary," and that, in others, induration and bubo, and probably secondary symptoms will occur, unless it be exhibited. If the first of these assertions is a mere matter of experience, and we think that the generality of surgeons are adverse to the position of Mr. Wallace, the second is a repetition of an assumption to which we have already referred. But what does Mr. Wallace mean, when he says that induration may follow gonorrhœa? Is it induration of the corpus spongiosum? If so, we can assure him that that is the result, the natural result, of inflammation extending to that structure, and certain we are it is no evidence whatever of specific action.

Taking the whole affair in the lump, we may safely say, in reply to Mr. Wallace, that he stands, and is likely still to stand, alone in his general recommendation of a course of mercury for gonorrhœa. We have seen it tried frequently, but we never yet saw the least satisfactory evidence of its utility. Here then we are toto cœlo arrayed against Mr. Wallace's opinions and advice. But to proceed with the treatment of this complaint. At the same time that Mr. W. is exhibiting mercury, he exhibits also cubebs and copaiba.

"At the same time that mercury is used in catarrhal syphilis, we are to administer such remedies as have a specific influence over the peculiar morbid action which especially distinguishes this disease—I mean copaiva and cubebs; and it is to be observed, that, as a combination of different purgatives will frequently act more beneficially and more effectually than any single purgative, the medicines just mentioned will also often act much more effectually if used in combination, than if given separately. Their power will also be increased by uniting them with such remedies as have an especial action on the urino-genital organs—as the aqua kali, nit. potassæ, spt. æther. nit.

Of the specific influence of copaiva and cubebs over the catarrhal form of primary syphilis, I can have no doubt. It will be, however, for the most part necessary to administer these medicines in larger doses than are commonly employed; and I consider it better to interrupt their employment every three or four days, for a day or two, than to give them uninterruptedly.

A state of severe passive inflammation forms no objection to the use of either cubebs or copaiva. Indeed, having premised evacuations, I have often employed them with great advantage, even when the inflammation seemed to be active. The medium dose of cubebs which I give, is six drachms in the day, and the medium dose of copaiva two drachms. These quantities are to be divided into three doses, and one dose given three times a day, about an hour after meals.

If either of these medicines has been given separately, and appears to disagree or to have lost its effect, much advantage will be derived from using the other—leaving off whichever may have been previously employed. Indeed I have, on some occasions, thought that it was more advantageous to alternate the use of the cubebs and copaiva with each other, than to give them combined."

Mr. Wallace proceeds to a little disquisition, of the nature that he loves, on stimulants and tonics and astringents; and he proves, as clearly as the nature of the thing will allow it to be proved, that a stimulant is not a stimulant, and that an astringent is something else. What a happy talent that must be, which can satisfy ourselves of the truth and soundness of our most recondite speculations: and can demonstrate with the precision of Philosopher Square, and admit with the faith of Parson Allright, not only the absolute fitness of things, but the divine rule of grace. That talent is possessed in an eminent degree by Mr. Wallace. His practice may not fit his theory, but his theory never fails to suit his practice. We will not be so ill-natured as to say, as Gulliver said of the tailors of Laputa, that although they cut their coats upon abstract principles, those coats were the worst made that he ever saw; but we must admit that Mr. Wallace's treatment is an olla-podrida of every notion and every mode of management, a Koran picked from Jew and Christian, in which every sect discovers at the same time that its tenets are adopted, and itself is damned.

We should like to quote the theoretical veiws of Mr. Wallace on the operation of stimulating applications; but our limited space must prevent us from indulging the natural desire, and we must content ourselves with stating that Mr. Wallace prefers employing the precise and definite designations of alterative and tonic, to the loose and improper term of stimulant. Thus fifteen grains of nitrate of silver dissolved in an ounce of distilled water, is his "tonic and alterative application," and one "of a medium strength"!

" The tonic or alterative application which I prefer in catarrhal primary syphilis, as long as there exists a high degree of morbid sensibility, is a solution of the nitrate of silver in distilled water. When this state of morbid sensibility is diminished, I use a solution of the muriate of mercury; and when all morbid sensibility has ceased, and a discharge still continues, a solution of the acetate of zinc, or of the sulphate of copper, &c., is employed. The strength of these injections must vary under different circumstances. Fifteen grains of the nitrate of silver to an ounce of water, half a grain of the muriate of mercury, or a grain and a half of the acetate of zinc, or of the sulphate of copper, to the same quantity of water, will form solutions of a medium strength."

" Should a state of morbid sensibility with discharge continue in the male urethra after the judicious and successive employment of the remedies above mentioned, great advantage will be very often derived from the introduction of the bougie or catheter, and from the application of the nitrate of silver in such a manner as to vesicate or excoriate the surface of the penis along the urethra from the scrotum forwards, when the disease seems to be confined to the front part of the penis; or from its application to the perinæum, when there is reason to believe that the disease has extended further towards the bladder."

We believe we have put our readers in possession of Mr. Wallace's views of gonorrhœa, and the principles of treatment which he recommends.—Those principles are somewhat subtle—that treatment rather complex. To the subtlety of the former we need make no farther allusion now, but we think we may venture to point out once more the composite quality of the latter. At one and the same instant the patient is to be subjected to a course of mercury, to take cubebs *and* copaiba, *and* to use an injection of nitrate of silver of the moderate strength of fifteen grains to the ounce of water. This heterogeneous mess of remedies of all descriptions, and seemingly of precisely opposite effects, would seem to defy a consistent theory. Yet a theory is offered by our author, and success is as boldly declared to attend them.

For our own parts, like Boswell, we admire, even if we cannot acquiesce. When Bozzy was staggered by any very violent dogma of his master, he gently admitted his own incapacity. "Ah! Doctor," he said, "the evidence that your mighty mind rejects is amply adequate for mine. What won't fill a quart pot, will fill a pint." In this instance we are forced to confess ourselves the pint pot, and the ordinary opinions on the nature and the management of gonorrhœa, fill us too completely to admit even the drippings from Mr. Wallace's quart.

As practical men, we are reduced to something like this moderate creed:—that we know of no means of distinguishing discharges from the urethra after connexion, from what is denominated gonorrhœa—that we have never seen unequivocal secondary symptoms after gonorrhœa, and although we do not deny their occurrence, we contend that that occurrence must be rare—that this is of itself a broad and a satisfactory distinction between gonorrhœa and syphilis—that, therefore, while we advocate a moderate and rational mercurial treatment for the latter, we cannot advocate and do not practise the exhibition of mercury for the former.

Perhaps we may be permitted to conclude this article, already rather long, with a rapid exposé of our own views of treatment. And as we have paid some attention to the subject, we trust we shall not be deemed impertinent in offering the opinions we have formed.

It is indispensable on the part of the surgeon to remember, that gonorrhœa is a compound of a specific complaint and of ordinary inflammatory action. In some cases inflammation is found to be considerable—in others it is not. Common sense whispers, and experience amply confirms its dictates, that the same treatment can scarcely be proper under such opposite circumstances. The complications of gonorrhœa are usually inflammatory, and consist of inflammation of tissues in immediate or remote connexion with the mucous membrane primarily implicated.

The great rule on which we would insist is first to remove the inflammatory action; secondly, to arrest the specific discharge. So long as there is violent pain in micturition, and decided purulent secretion, so long, we conceive, there is active inflammation.—This we should therefore attempt to subdue, as we would inflammation of any other mucous membrane, by smart doses of calomel with antimony and opium—active purges—starvation—rest—and diluents. If inflammatory action runs very high, local or even general depletion is required. Such is our treatment of the inflammatory stage of gonorrhœa. As soon as that stage is decreasing or has ceased, we immediately begin to exhibit copaiba or cubebs, combined with injections of alum or lead. These means are usually extremely successful, and what is more, they are extremely safe. Within these last twelve months we have treated a large number of cases of gonorrhœa in this way, and in not one case has hernia humoralis or any inflammatory complication supervened. We are satisfied of the safety of this plan of treatment.

We admit that pain in the act of micturition should not always preclude the exhibition of copaiba, or cubebs, and injections. When that pain persists, although the other inflammatory symptoms have subsided, it will often be removed by stimulating remedies. When the discharge is thin, when swelling of the penis has ceased, and when the pain is disposed to vary in its seat and its intensity, we have no hesitation in prescribing copaiba, or means of that description.

Persons who have frequently had gonorrhœa may certainly employ the stimulating treatment with more safety and success while symptoms of inflammation remain, than those who have not previously suffered from the malady. This is analogous to what we observe in other diseases and in other parts. The history of the patient should therefore be investigated, and the surgeon should always particularly inquire the circumstances attending former claps, and the remedy that previously agreed the best.

Cubebs certainly agrees much better with some persons than with others. On some it appears to exert no influence—on others, the minority, its effects are excellent. We have little hesitation in prescribing it as soon as the discharge is becoming thin, although there is yet much smarting during the act of micturition. But we think that the patient should be briskly purged, at the time that the cubebs is exhibited.

We cannot agree with those who use injections of the nitrate of silver, in the early stages of gonorrhœa. We have frequently employed them, seldom with advantage, sometimes with injury. We dread their use. But, in cases where other means are failing, and chronic gleety discharge is established, the nitrate is occasionally beneficial. We usually begin with a solution of two grains in an ounce of water, and increase the strength to one of five or six grains in the same quantity. This usually renders the discharge again purulent, and increases or re-establishes the scalding. We then turn round, discontinue the injection of the nitrate of silver, and substitute for it a solution of lead. This not unfrequently effects a cure.

We have not yet spoken of the inflammatory complications of gonorrhœa—inflammation and abscess of the cellular tissue of the penis—inflammation of the absorbents of the penis—bubo—inflammation of the lacunæ of the urethra—inflammation and induration of the corpus spongiosum—inflammation of the prostate, the bladder, or the testis. Some of these are familiar, and some are not. In the work of Mr. Wallace, (one meant to be elaborate,) several of these affections are not even noticed; a strange omission, equally observed in other works upon this disease. Our space will not permit us to pursue the subject farther at present, and for observations on some of the complications we have mentioned, we refer to two papers on the management of gonorrhœa, published by Mr. Henry James Johnson, in preceding numbers of this Journal.*

---

Extract of a Letter to the Editor, from Sir David J. H. Dickson, M.D. &c. Physician to the Royal Hospital, Plymouth, 18th May, 1835.

The following is some account of an interesting dissection of a case of phthisis which we lately met with, attended with pneumo-thorax, and displacement of the liver and stomach.

Mr. J. C. ætatis 30, assistant-surgeon, R.N. was admitted into this hospital, on the 3d of April, with symptoms indicating the last stage of phthisis, viz. great dyspnœa, cough, purulent expectoration, marasmus, &c. and died on the 29th of April, 1835.

*Sectio Cadaveris*, 28 *Hours post Mortem*. The right side of the thorax was evidently enlarged, and, upon opening it, a quantity of inodorous gas rushed from the cavity of the pleura. The lung, which was compressed into a small size, in the upper part of the cavity, lay close to the mediastinum and spine, to which it was firmly bound down posteriorly; while, at the same time, the fore-part was attached by two strong adhesive bands, passing to the anterior and upper part of the chest. The structure of the superior lobe was much condensed, and it was a mass of firm, crude tubercles, while, from the softening of those bodies in other places, the rest of the lung displayed several anfractuous excavations, traversed by bands, which seemed to consist of obliterated bloodvessels and indurated pulmonary tissue.

---

* Nos. 38 and 39.

In one of the largest of these vomicæ, in the inferior lobe, there was an irregular opening into the cavity of the chest, caused by ulceration of the pulmonary pleura. The base of the cavern was of a denser structure, and puckered, with whitish ridges or elevations intersecting it.

There was about a pint of sero-purulent fluid in the right cavity of the chest, produced, I presume, by partial pleurisy, and mixed with shreds of coagulable lymph; for, corresponding to the part in which the patient had complained of pain, some portions of the costal pleura were reddened, and coated with a layer of false membrane.

Pleural adhesions, but easily separated, had, also, taken place on the left side of the chest; the upper lobe was tuberculated, and the rest of the lung was much engorged with blood and serum. I may here notice that the patient (who, on his admission, still indulged the "delusive hope of recovery," though in the last stage of phthisis) could only lie upon his back, or for a very short time on the left side, and not at all upon the other; and he several times observed, when within a few days of his death, that he felt as if he only breathed from a small portion of the upper part of the left lung. The heart was normal.

From the great depression of the diaphragm, caused by the pressure of the gaseous and liquid effusions in the right cavity of the pleura, the convex surface of the liver was much flattened; and this organ, which was healthy, was pushed from the right towards the left side. But the stomach was displaced in a much more remarkable degree, being quite on the left side of the spine—in fact, it was found lying in the left hypochondrium longitudinally, and with its most depending portion almost extending to the superior border of the os ilii.*

The inner surface of the stomach presented a curious mammillated appearance, from numerous little prominences of the size of marbles, evidently caused by emphysematous infiltration of the submucous cellular tissue; for air escaped on puncturing them, and, by pressing these vesicular elevations with the fingers, they could be readily made to recede from one place, and to re-appear in another. Some portions of the jejunum, and much of the ileum, were injected, and of a deep red colour externally and internally; and the angry-looking, white, dotted patches on the outer, denoted, as usual, the seat of ulceration on the inner surface. Many of these ulcers were isolated, but the greater portion of the lower third of the ileum especially, where the patches of Peyer are situated, was abraded by numerous ulcerations, some of which had penetrated even to the peritoneal covering of the bowels. These ravages frequently extend beyond the ileo-cæcal valve, but they were not observed to have done so on the present occasion. For the large intestines were not diseased, though the transverse, and the descending colon especially, were much contracted.

Morbid appearances, though differing in degree, yet similar to those noticed above, supposed to arise from enlargement and chronic inflammation of the isolated and aggregated glands, or follicles of the mucous coat of the ileum, or from the deposition of tubercular matter in the submucous tissues, I have generally observed in the dissec-

* In one case of phthisis, I have found the stomach so remarkably enlarged and extenuated, that it nearly covered the whole of the intestines, and extended to the iliac fossa on each side, with the intermediate portion resting upon the pubes. It contained about a gallon of fluid, and, with a small portion of the duodenum, removed with the pylorus (which was dilated, instead of being obstructed) was capable of holding fourteen pints of water; but, with the exception of a small portion of the great curvature, it was so extremely attenuated, that the peritoneal coat alone seemed to remain, and it gave way on attempting to preserve it by inflation. Two instances, strikingly similar, are adduced in M. Andral's Memoir on Chronic Gastritis, inserted in the Number for January, 1827, of the Medico-Chirurgical Review, vol. x. p. 159.

tions of phthisical patients; and those, as well as various other lesions of the alimentary canal, to which I must not now diverge, met with in the post-mortem investigations at this hospital, are skilfully delineated in the excellent pathological works of Dr. Carswell and Dr. Hope.

The mesenteric glands, from tuberculous deposition, had acquired an extraordinary increase of size in the present instance; many of them were the bulk of a nutmeg, and others were as large as a walnut. They accurately resembled the clusters of enlarged glands in the first figure of the third plate, in the fasciculus on Tubercles, published by the former of these able pathologists.

David J. H. Dickson.

---

### Last Curriculum from the Society of Apothecaries.

We have given the whole of this long and complicated document in an Appendix, since it must prove generally interesting to a wide circle of our readers. Advocating, as we have always done, an extension of the course of education, classical, mathematical, and medical, we hail, with joy and approbation, every approach to the object of our hopes and wishes. It will be obvious that the Court of Examiners are every year approximating to the scale of education which we have so often urged on the profession. If our readers will turn back to our January Number for last year, page 280, they will there see what we have said respecting apprenticeships; and the present curriculum will shew that the Court of Examiners have *almost* become converts to the plan we sketched out in our April Number of last year, page 569. In that plan, we proposed that "the apprenticeship should be limited to two years." It will be seen that the Court has recommended the curtailment of the said apprenticeship to *two years and a half*—namely that the pupil should be liberated in the third year, in order to prosecute his studies in London or elsewhere. We recommended "three academic years of study." The Court has adopted this proposal to the very letter. There are to be, not only *three winter sessions* of study, but there are to be summer sessions, not previously enforced.

Wishing, as we do, to see the whole profession regulated and superintended by one supreme council or academy, we nevertheless give great praise to the Court of Examiners for thus, as it were, anticipating much of what that academy will be likely to enact. The Company have, by this curriculum, placed the general practitioner on a level with the physician, in point of solid medical education—and, indeed, much higher on the scale than the physicians were, even within the last 20 years. This procedure must tend very much to diminish the influx into the ranks of the profession, by increasing the expense and elevating the education. The members will necessarily be more select and respectable. Such selection and respectability, were no legal enactments to take place by Parliament, will, in no long time, change the present disgraceful system of having medical remuneration charged entirely on drugs. Those who have incurred the heavy expenses attendant on an apprenticeship, and three academic years of study, will surely not stoop to the degrading system of druggery now imposed on them. They will throw off the yoke, even if the legislature decline to emancipate them! But it is hardly possible that the Parliament, while reforming Corporations generally, will let the medical corporations pass unnoticed. The spirit of reform is abroad—and, whether the pressure be from within or from without, the medical polity will be squeezed into some decent and rational shape before the expiration of two years from this time. *

---

* We could offer objections to some of the details of the present Curriculum, and especially to the plan of specifying the exact number of lectures, of which a course shall consist. This is too much like *weighing* rhubarb or jalap, in the shop of the Hall in Bridge-street.—Eds.

**REMARKABLE CASE OF EXTENSIVE AND COMPLICATED ORGANIC DISEASE, ILLUSTRATING THE SYMPTOMS DURING LIFE. By R. BILLIS, Esq. Surgeon, Maidenhead.**

[☞ *This case came once under our own observation, and the symptoms were in strict accordance with those stated by Mr. Billis.—Ed.*]

By referring to our day-book,* I find our first attendance on Mr. S. was in May, 1832. He then laboured under general derangement of the chylopoietic viscera, which yielded in a few days to the usual treatment.

In the following July, he had the misfortune to break two or three of his ribs, and, although Mrs. S. informs us that Mr. S. never considered himself well after this accident, we did not see him again, professionally, till July, 1833, when we found him suffering from the following symptoms:—Loss of voice, difficulty of breathing, excessive pain in the back and shoulders, no appetite, &c. He only remained under our care for ten days, nor did he consult us again till last June. For some months prior to this, his health had been very indifferent, and he had consulted several medical men in London and Bath. His case now bore a very serious aspect; the symptoms above enumerated were very much aggravated, and the arterial system greatly deranged, palpitations, &c. being of frequent occurrence. Although these were present, more or less, in the previous attacks, we certainly did not consider them as indicating organic disease, but looked upon them as merely sympathetic. But now, having the patient constantly under our observation, we soon became convinced that there was organic disease, either of the heart or the large vessels. At this time he suffered very much from sickness, and alternate diarrhœa and constipation; the former ceased in a few weeks, but the latter continued till death, requiring the constant use of lavements. There were frequent sanguineous discharges from the bowels, and occasionally substances somewhat resembling pieces of wax candles. Such is the history of the case up to January last, when he consulted Dr. Johnson. From that time his strength gradually declined, the symptoms continuing much the same, excepting the difficulty of breathing, which, a few days prior to his death, became distressingly painful, both to himself and those around him. The pulse at the left wrist could not be felt for a considerable time. The expectoration was small in quantity, and decidedly mucous. He died on the 20th April, æt. 50. I am informed that, 14 years ago, he experienced a severe attack of acute rheumatism.

The treatment may be summed up in a few words:—Absolute rest, both mental and corporeal, was particularly recommended, but the patient's anxiety about his affairs prevented this being strictly enforced, as Mr. S. frequently transacted business, not only in his bed-room, but in bed, where he might often be seen surrounded by all the paraphernalia of an attorney's office. A light mild diet, and medicines to allay the more urgent symptoms, such as aperients, antacids, sedatives (particularly hydrocyanic acid and digitalis), and anodynes were prescribed. He had accustomed himself to considerable quantities of opium.

*Dissection.* The body was very much emaciated, but did not present anything else externally worth noticing. The pleura pulmonalis and pleura costalis of the right side, were completely glued together throughout their whole extent: the left pleura contained a considerable quantity of serous fluid, but no fibrine. On the external surface of the *inferior* portion of the right lung was a large quantity of a whitish soft matter, (tuberculous infiltration,) of the consistence of soft brain: and separating this from the substance of the lung, was an ossific partition, rather more than two inches by one in extent, and a line in thickness.

The left lung was completely studded with very hard granulations, and here and there a tubercle in the crude state, but none absolutely broken down. The lungs generally were so much congested as to present the appearance of being

* Messrs. Bishop and Billis.

very much larger than natural: they were also (especially the right) loaded with mucus. The pericardium contained several ounces of fluid, and the heart was decidedly enlarged. There was a small osseous deposit in the base of one of the semilunar valves. There was an aneurism of the arch of the aorta which measured twelve inches in circumference, and another on the right side of the descending aorta, about the size of an egg. These (after macerating them in water, so as to completely remove the blood) I examined with considerable care. A considerable portion of the large aneurism was filled with coagulated lymph, and to the margin of this, but no farther, could the internal and middle coats of the aorta be traced. The fibrine being entirely removed, the external coat could be distinctly seen deprived of the middle and internal ones, but of extraordinary tenuity. The small aneurism was a good specimen of the true species, the three coats being entire over every part of it.

On the right side of the posterior portion of the aneurism it was firmly united to the trachea, and so thin was the separation between the interior of the one and the interior of the other, that the slightest touch with my finger made a communication between them.

I will just observe here that the patient appeared to die from suffocation.

I ought to have observed above, that the fibrine, when removed from the tumor, weighed six ounces, and was three quarters of an inch in thickness. The orifice of the left subclavian was completely plugged up. The coats of the stomach and bowels were remarkably pale and attenuated. There were currants in the stomach which had been taken more than three weeks before death. There was a crude tubercle in the liver, and the spleen was small, and of a leaden hue.

It affords me great pleasure in being able to state that the prejudice against post-mortem examinations is fast subsiding. I have not met with a single refusal during the last three years, and in that time I have instituted at least a dozen, which, for a country practitioner, you must allow, is a tolerable number.

The result of the examination in this case was very satisfactory, as it justified the view we took of it, and confirmed your opinion as to the existence of an aneurism. Most of the leading symptoms may now be easily accounted for. The loss of voice by the pressure of the aneurism on the trachea; the dyspnœa, by its compressing the thoracic contents, and also by the disease of the left lung; the want of pulsation in the left radial artery by the plugging up of the left subclavian. The pain in the back and shoulder I confess my inability to explain; as the spine was sound, and if the pain had been caused by pressure on the brachial plexus, the arm would also have suffered. Vomiting and purging, in the early stage, we considered (judging from the state of the evacuations) occasioned by vitiated bile; and the constipation which succeeded was owing partly to there being a deficiency of bile, (the liver was very small, and appeared to have been inactive,) and partly to the opium. The blood from the bowels, I imagine, must have been caused by piles, (no external appearance of which could be discerned,) or arterial exhalation; and might not the substances, which I have described as resembling wax candles, have been tuberculous matter, secreted by the mucous membrane of the bowels?

B. Bellis.

---

Case of apparently Secondary Symptoms after Gonorrhœa. By H. J. Johnson, Esq,

The rarity of secondary symptoms as a consequence of gonorrhœa has been dwelt on at some length in a preceding article. I hardly know whether to bring forward the present case as an instance of the occurrence of such symptoms, or not. The case itself is yet in progress, and no definite conclusions of any kind can be drawn from it.

Eight months ago, a gentleman placed himself under my care on account of gonorrhœa. There were only slight inflammatory symptoms, and it seemed to be a simple and a mild attack. He had once previously suffered from the complaint. I need not mention the treatment employed, as it merely consisted of the ordinary remedies. Gleet, however, succeeded to the purulent discharge and scalding, and upwards of two months elapsed before this had completely disappeared.

Two months ago, three or four months after all the symptoms of gonorrhœa had ceased, a bubo formed in the left groin, and in spite of all remedies and the utmost care, it advanced to suppuration. I opened it with the lancet, and the wound slowly healed.

About a month ago, the bubo being then open, the mucous membrane of the lower lip at its commissure with the gum, and the gum itself, opposite the third molar tooth on the right side of the lower jaw, exhibited each a small yellow ulceration, resembling what mercury sometimes produces. The ulcer was hollow, yellow on the surface, and exhibited a thin, rather undermined edge. The throat felt sore, but no distinct ulceration was perceived in it. These ulcers were slowly healed by the application of the lunar caustic, when, suddenly, the right knee and left elbow were attacked with acute synovial inflammation accompanied with much effusion in the joint.

The patient's condition at present is this. There is slight ulceration still in the gum, but none on the mucous membrane of the lip. The bubo is quite healed. There is not, and there has not been since the disappearance of the discharge, five or six months ago, the slightest symptom of any thing wrong in the urethra. The synovial inflammation of the knee and elbow has abated, but is still severe.

The patient's health is but indifferent, and he seems to have been rather addicted to intemperance.

Such are the principal facts of this case. I should observe that the patient has taken no regular mercurial course, but I gave him mercurial purgatives freely in the early stage of the gonorrhœa. After the bubo was laid open, he was ordered sarsaparilla in combination with the liquor potassæ.

Is this to be regarded as an instance of secondary symptoms after gonorrhœa; or may those symptoms be deemed the effects of impaired constitutional power? They certainly present features very different from those of secondary syphilitic symptoms; for in syphilis, bubo is commonly an early symptom, ulcers seldom occur in the mouth independently of other ulcerations in the throat, and synovial inflammation is chiefly observed in persons broken down by the injurious operation of mercury. On the other hand, it is difficult to believe that such phenomena as occurred in the case I have related, have merely had their source in a bad state of health. Such effects are too rarely seen from such a cause to induce us to assent readily to such a supposition.

The observations of Sir Benjamin Brodie are sufficient to establish the occasional occurrence of synovial inflammation, in connexion with gonorrhœa. Yet so far as I have seen, the cases that occurred to that able surgeon, were more severe than they usually are. Within this last year I have seen three instances of this description. In one the ankles and one knee were attacked—in the second the left knee was the seat of inflammation—and in this, the third instance, the affection has hitherto been limited to one elbow and one knee. The first patient speedily recovered under colchicum and blisters—the second was confined to his bed for a month, and then recovered completely.* The third is still labouring under the complaint, which, however, appears very far from intractable. In the two first patients there was inflammation of the conjunctiva of both eyes, but in neither was much purulent discharge established.

Leeches, blisters, calomel and opium, and saline purgatives with colchicum, have appeared in these and in other cases that have fallen under my observation, to exert almost as great an influence, as in ordinary rheumatic inflammation. At all events they removed the disease in a moderate period of time.

---

* This patient had purulent ophthalmia in connexion with a former gonorrhœa; and his brother had suffered severely under gonorrhœal rheumatism.

